# Novel Snapshot-Based Hyperspectral Conversion for Dermatological Lesion Detection via YOLO Object Detection Models

**DOI:** 10.3390/bioengineering12070714

**Published:** 2025-06-30

**Authors:** Nan-Chieh Huang, Arvind Mukundan, Riya Karmakar, Syna Syna, Wen-Yen Chang, Hsiang-Chen Wang

**Affiliations:** 1 Diving Medical and Physiology Training Center, Zuoying Armed Forces General Hospital, No. 553, Junxiao Rd., Zuoying District, Kaohsiung City 813204, Taiwan; pippen0050@gmail.com; 2 Department of Information Engineering, I-Shou University, No.1, Sec. 1, Syuecheng Rd., Dashu District, Kaohsiung City 84001, Taiwan; 3 Department of Mechanical Engineering, National Chung Cheng University, 168, University Rd., Min Hsiung, Chia Yi 62102, Taiwan; arvindmkukund96@gmail.com (A.M.); karmakarriya345@gmail.com (R.K.); 4 Department of Biomedical Imaging, Chennai Institute of Technology, Sarathy Nagar, Chennai 600069, India; 5 Department of Computer Science and Engineering, Chitkara University, Chandigarh-Patiala National Highway NH-64 Village Jansla, Rajpura 140401, India; synab6498@gmail.com; 6 Department of General Surgery, Kaohsiung Armed Forces General Hospital, 2, Zhongzheng 1st. Rd., Kaohsiung City 80284, Taiwan; 7 Department of Medical Research, Dalin Tzu Chi Hospital, Buddhist Tzu Chi Medical Foundation, No. 2, Minsheng Road, Dalin, Chiayi 62247, Taiwan; 8 Hitspectra Intelligent Technology Co., Ltd., Kaohsiung 80661, Taiwan

**Keywords:** skin cancer, hyperspectral imaging, spectroscopy, YOLO, machine learning, narrowband imaging

## Abstract

**Objective**: Skin lesions, including dermatofibroma, lichenoid lesions, and acrochordons, are increasingly prevalent worldwide and often require timely identification for effective clinical management. However, conventional RGB-based imaging can overlook subtle vascular characteristics, potentially delaying diagnosis. **Methods:** A novel spectrum-aided vision enhancer (SAVE) that transforms standard RGB images into simulated narrowband imaging representations in a single step was proposed. The performances of five cutting-edge object detectors, based on You Look Only Once (YOLOv11, YOLOv10, YOLOv9, YOLOv8, and YOLOv5) models, were assessed across three lesion categories using white-light imaging (WLI) and SAVE modalities. Each YOLO model was trained separately on SAVE and WLI images, and performance was measured using precision, recall, and F1 score. **Results:** Among all tested configurations, YOLOv10 attained the highest overall performance, particularly under the SAVE modality, demonstrating superior precision and recall across the majority of lesion types. YOLOv9 exhibited robust performance, especially for dermatofibroma detection under SAVE, albeit slightly lagging behind YOLOv10. Conversely, YOLOv11 underperformed on acrochordon detection (cumulative F1  =  65.73%), and YOLOv8 and YOLOv5 displayed lower accuracy and higher false-positive rates, especially in WLI mode. Although SAVE improved the performance of YOLOv8 and YOLOv5, their results remained below those of YOLOv10 and YOLOv9. **Conclusions:** Combining the SAVE modality with advanced YOLO-based object detectors, specifically YOLOv10 and YOLOv9, markedly enhances the accuracy of lesion detection compared to conventional WLI, facilitating expedited real-time dermatological screening. These findings indicate that integrating snapshot-based narrowband imaging with deep learning object detection models can improve early diagnosis and has potential applications in broader clinical contexts.

## 1. Introduction

Skin cancer is a major global health concern, with incidence rates steadily rising over recent decades. According to the World Health Organization, skin cancer accounts for approximately one-third of all cancers worldwide. In 2022, an estimated 115,320 new cases of skin cancer (excluding basal and squamous) were reported, alongside around 11,540 deaths [1]. Mortality rates for skin lesions vary, with men generally exhibiting higher overall mortality than women [2]. Dermatofibromas (DFs), lichenoid keratosis, and acrochordons are less common types of skin lesions, each accounting for less than 10% of cases [3]. DFs with classical morphology have been observed in children under the age of five [4]. Lesions larger than 1 cm with positive surgical margins show a recurrence probability of approximately 10%, which is lower than previously reported rates ranging from 26% to 50% [5]. DFs typically develop on the lower body but can also appear in various locations, including the head, face, auricle, neck, trunk, shoulder, pelvic girdles, and fingers [6,7,8,9]. They are more frequently diagnosed in women, with a lifetime prevalence estimated between 1% and 2% [10]. Lichenoid eruptions occur in children and arise from various causes. Despite the lack of a definite cause, immune-related mechanisms are believed to play a central role [11]. Owing to their typically asymptomatic nature and frequent misdiagnosis, the precise incidence rates of lichenoid eruptions are not well documented. Skin tags, clinically known as acrochordons, are small, irregularly shaped growths commonly found in skin folds [12]. Approximately 46% of the population develop skin tags, though severe cases are rare [13]. These lesions are especially prevalent among obese individuals and patients with type II diabetes, affecting roughly one-quarter of the adult population [14,15].

DFs are firm, reddish to brown nodules mainly found on the distal limbs [16]. Histologically, these nodules comprise fibroblasts, histiocytes, and collagenosis. However, DFs can sometimes mimic malignant lesions due to their firmness and coloration [17]. While typically asymptomatic, patients may occasionally experience itching or tenderness in the affected area. Lichenoid keratosis primarily presents as several erythematous-brown, flat-topped papules or slightly raised, reddish-brown, round or oval, flat-topped plaques. Histopathologically, this lesion features basal layer hyperplasia resembling lichen planus (LP), degeneration of basal keratinocytes, and band-like distribution of estrogen receptor-positive lymphocytes. Lichenoid lesions refer broadly to papular lesions observed in several dermatologic conditions, with LP being a notable example. These papules are shiny, flat-topped, polygonal, and clustered, often likened to lichen growing on rocks. Histologic examination reveals inflammatory cell infiltration arranged in a dense, band-like pattern that obscures the dermo–epidermal interface [11]. Acrochordons, or skin tags, are asymptomatic, skin-colored, frustum-shaped growths commonly found in skin folds, particularly in the neck, axillae, and groin regions. They comprise loose, fibrous connective tissue covered by an epidermal layer [18]. Although benign, acrochordons are susceptible to irritation due to their frequent occurrence in areas subject to friction or trauma [19]. Unlike malignant skin lesions such as melanoma and non-melanoma carcinomas—which demonstrate cytologic atypia, invasive growth, and metastatic potential—dermatofibromas, lichenoid keratosis, and acrochordons lack these aggressive features. Therefore, the three target categories are classified as benign lesions in the SAVE-enhanced imaging and the subsequent YOLO-based detection tasks.

Imaging spectroscopy, also known as hyperspectral imaging (HSI), refers to the measurement, analysis, and interpretation of spectral data from images [20]. HSI is an advanced imaging modality that captures detailed information on the pathological and molecular characteristics of tissues, insights that are often difficult to obtain from routine diagnostic imaging methods [21]. This technique operates by capturing hyperspectral data across hundreds of contiguous wavebands, offering a rich, multilevel representation of the target area [22]. The availability of crucial spectral information allows HSI to distinguish between mucosal and submucosal features that are invisible to the human eye within the white light imaging (WLI) optical range [23]. Unlike traditional imaging, HSI constructs a data hypercube by recording a spectrum at every spatial resolution cell. HSI cameras can capture light across a wide spectral range, including ultraviolet bands ranging from 200 nm to 380 nm, visible light bands ranging from 380 nm to 780 nm, and near-infrared bands ranging from 780 nm to 2500 nm [24,25]. Therefore, HSI has been applied in several fields of study, including astronomy [26], agriculture [27], molecular biology [28], medical imaging [29,30], mineralogy [31], archeology [32], the food industry [33], and environmental studies [34], due to its versatility and rich spectral output.

Narrowband imaging (NBI) is an HSI technique that uses narrow wavelength bands to enhance certain tissue features required for medical diagnosis. This method employs two narrow beams of light: one in the blue spectrum at 415 nm and the other in the green spectrum at 540 nm [35]. These wavelengths enhance optical images by improving the visibility of superficial mucosal layers and submucosal intrapapillary capillary loops. The 415 nm wavelength enhances and highlights superficial vessels, while the 540 nm wavelength enhances deeper submucosal vessels, typically rendering them in cyan and brown hues, respectively. This special contrast enhances the sharpness between vessels and surrounding mucosa [36], resulting in the clearer visualization of shallow surface features, mucosal patterns, and vascular structures [37]. Band selection enhances the visibility of early-stage SC by providing clearer visualization of vascular patterns and superficial structures, compared to other imaging methods. When comparing WLI and band selection for detecting malignant features in skin lesions, band selection has demonstrated higher sensitivity, specificity, and overall accuracy [38,39]. While MSI and HIS systems offer detailed spatiospectral tissue characterization, their dependence on bulky scanning apparatuses or costly tunable filters limits their widespread clinical implementation. In endoscopic applications, pseudo-HSI technologies, such as Pentax i-Scan and Fujinon FICE, apply proprietary lookup tables to RGB sensor data. However, these systems often lack transparency and necessitate vendor-specific platforms, reducing flexibility. Although NBI is extensively validated and utilizes well-defined hemoglobin absorption peaks to enhance vascular detail, it generally necessitates specialized or modified light sources.

The prompt and accurate identification of skin lesions is crucial for improving patient outcomes. However, traditional RGB imaging often fails to adequately capture the subtle vascular patterns associated with the initial stages of lesion development. Although deep learning-based object detectors have seen rapid advancements, they are typically trained on standard color images, limiting their sensitivity to intricate capillary networks. Given the increasing prevalence of dermatological disorders and the high cost of clinical imaging devices, the necessity for an accessible and economical approach to improve vascular contrast without requiring a specialized apparatus is increasing. Therefore, this study introduces a novel approach using spectrum-aided vision enhancer (SAVE) technology. SAVE converts WLI into representations similar to NBI, comparable to systems developed for Olympus endoscopes, thereby enabling a clearer visualization of SC lesions. The proposed work integrates SAVE with advanced imaging modalities, employing machine learning methods (which include YoloV11, Yolov10, YoloV9, YOLOv8, and YoloV5) to develop an SC detection system targeting three lesion types: dermatofibroma, lichenoid keratosis, and acrochordon. The SAVE methodology addresses the limitations of standard RGB imaging by converting RGB images into narrowband-like representations that emphasize hemoglobin absorption characteristics, thus offering enhanced input to cutting-edge YOLO-based detectors. The principal contributions of this manuscript are as follows:

- SAVE: a snapshot-based technique that transforms any RGB image into a narrowband representation aligned with hemoglobin absorption peaks.
- Integration of SAVE with YOLO: adaptation of YOLOv5–YOLOv11 by incorporating SAVE outputs for real-time skin lesion detection.
- Comprehensive Evaluation: an exhaustive comparison of five YOLO variants across WLI and SAVE modalities, reporting class-specific precision, recall, F1 score, and statistical significance.

## 2. Materials and Methods

### 2.1. Dataset

The images used in this study were sourced from the publicly available International Skin Imaging Collaboration (ISIC) archive, which offers dermoscopic and clinical photographs annotated by experts. For the experiments, the following three distinct types of skin lesions were selected:

- Acrochordon: 577 images;
- Dermatofibroma: 821 images;
- Lichenoid lesions: 805 images.

All ISIC images originally had varying resolutions, ranging from 800 × 600 to 2048 × 1536 pixels. Aiming to standardize the input for the deep learning models, all images were resized to 640 × 640 pixels before training and inference. The SAVE method converted each three-channel RGB image into a five-band NBI approximation in a single snapshot. The five output bands corresponded to the central wavelengths described in Section 2.2. Therefore, each SAVE-processed image of size 640 × 640 was represented as a 5 × 640 × 640 tensor. For comparison, WLI inputs were retained as standard 3 × 640 × 640 tensors (RGB). The full dataset of 2203 labeled images was partitioned into training, validation, and test sets using a 70%/20%/10% ratio, respectively, ensuring a proportional representation of each lesion class in all subsets. Table 1 below summarizes the exact image counts. A training set of 1542 images was used to optimize model weights. A validation set of 441 images was used to tune hyperparameters (e.g., learning rate, confidence thresholds) and apply early stopping. Meanwhile, a test set of 220 images was held out entirely during model development and was used only once at the end to report final performance metrics, as shown in Table 1. Experiments on data augmentation techniques (random 90° rotations, horizontal/vertical flips, and small shear transformations) were also conducted. However, extensive augmentation introduced slight overfitting and did not improve validation accuracy. Consequently, all experiments reported herein were conducted using the original, non-augmented images.

### 2.2. SAVE

According to this study, converting RGB digital camera images into hyperspectral representations was efficiently achieved using the VIS-HSI conversion method introduced in this novel algorithm. While conventional NBI relies on specialized illumination at specific hemoglobin absorption peaks, the SAVE module learned a regression-based mapping from standard RGB camera responses to five designated center wavelengths (450, 500, 550, 600, and 650 nm). These wavelengths were selected based on the intersection between the spectral sensitivity curves of commercial RGB sensors and the documented absorption spectra of oxy- and deoxy-hemoglobin. This alignment ensured that the SAVE output improved vascular contrast similarly to authentic NBI. Although SAVE did not directly quantify tissue reflectance, this module empirically replicated the relative contrast between blood-rich regions and surrounding tissues, enabling cost-effective, NBI-like enhancement using any standard RGB image. While numerous manufacturers currently provide pseudo-HSI image enhancement, NBI was selected as the conceptual benchmark due to its extensive research backing and clinical validation as the leading technique for real-time vascular contrast enhancement in endoscopy. NBI uses well-defined narrowband illuminations centered on hemoglobin absorption peaks, offering a strong physiological foundation and extensive operator familiarity, in contrast to proprietary, device-specific lookup tables used by other systems. By aligning the spectral bands of SAVE with the target wavelengths of NBI, the proposed method was guaranteed to not only mimic the vascular contrast mechanisms trusted by clinicians but was also expected to remain fully compatible with standard RGB cameras, eliminating the need for specialized hardware or proprietary processing pipelines. During system calibration, data from a spectrometer were compared against corresponding RGB values using the Macbeth color checker (x-rite classic). The raw pixel data were normalized and linearized to produce accurate RGB values in the sRGB color space. A nonlinear correlation matrix and transformation coefficients were then applied to convert these values into the CIE 1931 XYZ color space. More specifically, the R, G, and B values (0 to 255) in sRGB were first reduced to a 0–1 range. A gamma correction function was applied to convert sRGB to linear RGB values. After error correction, the final X, Y, and Z values were updated (XYZ_correct_) and calculated using Equations (1) and (2).

(1) $$\left[C\right]=\left[{XYZ}_{Spectrum}\right]\times pinv\left(\left[V\right]\right),$$

(2) $$\left[{XYZ}_{Correct}\right]=\left[C\right]\times [V].$$

The integrals were then used to convert the reflectance spectra measured by the spectrometer into the XYZ color space.

(3) $$X=k\int_{400nm}^{700nm}S\left(\lambda \right)R\left(\lambda \right)\bar{x}\left(\lambda \right)d\lambda ,$$

(4) $$Y=k\int_{400nm}^{700nm}S\left(\lambda \right)R\left(\lambda \right)\bar{y}\left(\lambda \right)d\lambda ,$$

(5) $$Z=k\int_{400nm}^{700nm}S\left(\lambda \right)R\left(\lambda \right)\bar{z}\left(\lambda \right)d\lambda ,$$

(6) $$k=100/\int_{400nm}^{700nm}S\left(\lambda \right)\bar{y}\left(\lambda \right)d\lambda .$$

Multivariate regression analysis was employed to generate the correction coefficient matrix C. Using the reflectance spectrum data (R_spectrum_), the transformation matrix (M) corresponding to the colors on the X-Rite ColorChecker board was calculated. Aiming to reduce dimensionality and extract dominant spectral features, principal component analysis was applied to R_spectrum_, resulting in six notable principal components (PCs) and their associated eigenvectors. The six PCs accounted for 99.64% of the total variance, effectively capturing the essential spectral information. The resulting analog spectrum, denoted as ${\left[S_{\mathrm{Spectrum}}\right]}_{380–780nm}$, was reconstructed using Equations (7) and (8).

(7) $$\left[M\right]=\left[Score\right]\times pinv(\left[V_{Color}\right]),$$

(8) $${\left[S_{\mathrm{Spectrum}}\right]}_{380\sim 780nm}=\left[EV\right]\left[M\right][V_{Color}].$$

An average RMSE of 0.056 was computed across all bands from 380 to 780 nm, demonstrating the high accuracy of the spectral reconstruction. Before calibrating the camera, the mean chromatic aberration across all 24 color blocks was 10.76. This value decreased to 0.63 after calibration, indicating the improved accuracy of the vectorized kernels. A qualitative analysis was conducted to assess the color match in the LAB color space. The results showed that the colors were similar, with an average color difference of 0.75, supporting the effectiveness of the algorithm in converting RGB images to HSI images. The SAVE method presented in this study provides a detailed method for calibrating the NBI of the Olympus endoscope based on the HSI conversion algorithm (Supplement Figure S20 for the flowchart of the VIS-HSI imaging algorithm). Aiming to initiate the simulation process, the output images from the conversion algorithm were compared to realistic NBI images obtained using the Olympus endoscope (Supplement Figure S21 for the lighting spectrum difference between the Olympus WLI, Olympus NBI, and the Capsule WLI). The CIEDE 2000 color difference [40] was measured and reduced for each of the 24 color blocks. After correction, the average color difference across the blocks decreased to 2.79. Three main factors affected the accuracy of the color match. The first was the range of light wavelengths, or the light spectrum. The second was the color matching function, a mathematical function that quantifies the amount of light input at a specific color. The third factor was the reflection spectrum, which provides information regarding the amount of light reflected by a specific hue [34]. The illumination spectra of WLI and NBI were corrected using the Cauchy–Lorentz distribution, as shown in Equation (9), to reduce errors arising from differences in these spectra, particularly in the 450–540 nm region, where hemoglobin absorbance was high (Supplement Figure S22 for the difference between the Olympus SAVE and the VCE-simulated NBI lighting): (a) shows the difference between Olympus SAVE and Olympus WLI, and (b) shows the difference between VCE SAVE and VCE WLI images. The actual NBI images captured by the Olympus endoscope contained not only green and blue hues but also different shades of brown corresponding to a wavelength of approximately 650 nm, despite the peak absorption wavelengths of hemoglobin occurring at 415 and 540 nm (Supplement Figure S17 for the comparison of SSIM between the simulated NBI images and the WLI images of VCE and Olympus; Table S1 for the SSIM of 20 randomly chosen images in VCE and the Olympus endoscope). This finding indicates that nuanced image post-processing techniques contribute to highly realistic NBI images (Supplement Figure S18 for the comparison of entropy between the simulated NBI images and the WLI images): (a) shows the entropy for Olympus endoscopy, and (b) shows the entropy for the VCE camera. Table S2 presents the entropy comparison of WLI and NBI images in Olympus and VCE endoscopes. Therefore, in addition to the wavelengths of 415 and 540 nm, three additional spectral regions at 600, 700, and 780 nm were observed in this study (Supplement Figure S19 for the PSNR comparison of the 20 randomly chosen images in Olympus and VCE; Table S3 for the comparison of the corresponding PSNR values).

(9) $$f\left(x;x_{0},\gamma \right)=\frac{1}{\pi \gamma \left[1+{\left(\frac{x-x_{0}}{\gamma }\right)}^{2}\right]}=\frac{1}{\pi }\left[\frac{\gamma }{{\left(x-x_{0}\right)}^{2}+\gamma^{2}}\right]$$

**Figure 1:**
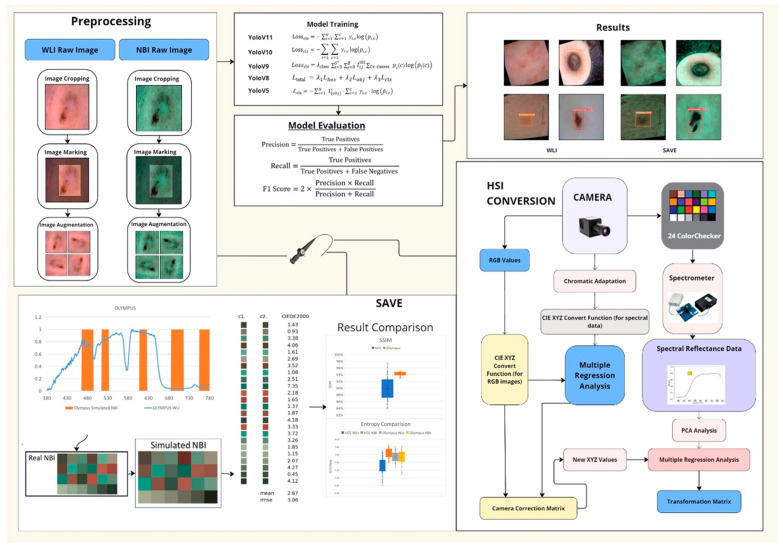
Schematics of the entire study.

Therefore, the optimization function used in this study was the dual annealing mechanical method, which enhanced the simulated annealing algorithm by combining elements of classical and fast simulated annealing with local search parameters to finetune the light spectrum [35]. A slight average standard CIEDE 2000 color difference of 3.06 was observed across the 24 colors. The overall flowchart of the project is shown in Figure 1 (Supplement Figure S20 for endoscopic imaging using three imaging techniques: (a) WLI, (b) NBI, and (c) SAVE).

### 2.3. ML Algorithms

All YOLO variants were trained in PyTorch 1.10.0 on a single NVIDIA V100 GPU (16 GB VRAM). Each model was optimized for 300 epochs with a batch size of 32 via SGD (momentum 0.937, weight decay 0.0005). The learning rate was initialized at 0.005 and annealed to 1 × 10^−4^ using a cosine schedule with a 3-epoch linear warmup. The validation F1 score was evaluated every epoch, and early stopping was triggered after 75 epochs without improvement, with the best checkpoint retained for final testing.

#### 2.3.1. YoloV11

YOLOv11 introduces several architectural improvements for real-time object detection, enhancing accuracy and speed [41]. This algorithm features a multi-path ConvNeXt backbone for improved feature extraction and incorporates dynamic convolutional attention (DCA) for highly effective object localization and classification. Additionally, YOLOv11 employs cross-stage partial connections to facilitate improved gradient flow in deep models without notably increasing computational cost. The adaptive anchor-free head replaces predefined anchor boxes for object detection, while the four-level prediction head improves detection across various object scales. For classification loss, YOLOv11 uses binary cross-entropy loss for multiclass classification.

(10) $${Loss}_{cls}=-\sum_{i=1}^{N}\sum_{c=1}^{C}y_{i,c}log\left(p_{i,c}\right)$$

Objectness loss uses logistic regression to estimate the confidence that a predicted bounding box contains an object.

(11) $${Loss}_{loc}=\sum_{i=1}^{N}\left[1-\frac{IoU\left(b_{i},{\hat{b}}_{i}\right)}{GIoU\left(b_{i},{\hat{b}}_{i}\right)}\right]$$

The improvements added to YOLOv11 make it one of the most efficient and accurate real-time object detection models.

#### 2.3.2. YoloV10

YoloV10 is a version of YOLO models that offers enhanced accuracy and fast processing times [42]. Aiming to enhance feature extraction, this version incorporates an upgraded backbone network and integrates attention mechanisms. The loss function of YoloV10 comprises the following three primary components: localization, objectness, and classification losses. For accurate class prediction, the classification loss is computed using binary cross-entropy. Using logistic regression, the objectness loss determines a confidence value for each anticipated bounding box.

(12) $${Loss}_{obj}=-\sum_{i=1}^{N}\left[y_{i}\log\left(p_{i}\right)+\left(1-y_{i}\right)\log\left(1-p_{i}\right)\right].$$

YoloV10 uses a generalized IoU loss to improve the localization loss through the elimination of bounding box coordinate inconsistencies and overlap measurements between predicted and ground truth boxes where the generalized intersection over union is represented by GloU. These enhancements help YoloV10 achieve high performance in real-time object detection.

#### 2.3.3. YoloV9

Two key innovations introduced by YOLOv9 in the field of real-time object detection are the generalized efficient layer aggregation network (GELAN) and programmable gradient information (PGI) [43]. PGI enhances model training by computing gradients through an auxiliary reversible branch. In addition to the operational methods discussed above, GELAN increases the number of parameters while maintaining high computational efficiency. This approach is achieved by building on the concepts of CSPNet and integrating ELAN. The corresponding loss function is defined in Equation (13).

(13) $${Loss}_{total}=\lambda_{cls}{Loss}_{cls}+\lambda_{loc}{Loss}_{loc}+\lambda_{obj}{Loss}_{obj}.$$

Bounding box regression loss is crucial for precise bounding box predictions and typically employs a mean squared error (MSE) approach, as shown in Equation (14).

(14) $${Loss}_{bb}=\sum_{i=0}^{N}\lambda_{\mathrm{coord}}\cdot \left[I_{i}^{obj}\right]\cdot \left({\left(x_{i}-{\overset{ˆ}{x}}_{i}\right)}^{2}+{\left(y_{i}-{\overset{ˆ}{y}}_{i}\right)}^{2}+{\left(\sqrt{w_{i}}-\sqrt{{\overset{ˆ}{w}}_{i}}\right)}^{2}+{\left(\sqrt{h_{i}}-\sqrt{{\overset{ˆ}{h}}_{i}}\right)}^{2}\right).$$

The confidence of the model in the presence of an object within a bounding box is measured using the objectness loss, which also helps determine the confidence score. The definition of the confidence loss function is presented in Equation (15).

(15) $${Loss}_{obj}=\lambda_{noobj}\sum_{i=0}^{S^{2}}\sum_{j=0}^{B}I_{i,j}^{noobj}{\left(c_{i}-{\overset{ˆ}{c}}_{l}\right)}^{2}+\lambda_{obj}\sum_{i=0}^{s^{2}}\sum_{j=0}^{B}I_{i,j}^{obj}{\left(c_{i}-{\overset{ˆ}{c}}_{l}\right)}^{2}.$$

Classification loss is a criterion that ensures the model correctly identifies the detected items by evaluating the accuracy of class regression using cross-entropy. The definition of the classification loss function is provided in Equation (16).

(16) $${Loss}_{cls}=\lambda_{class}\sum_{i=0}^{S^{2}}\sum_{j=0}^{B}I_{i,j}^{obj}\sum_{C\in classes}p_{i}\left(c\right)\log\left({\overset{ˆ}{p}}_{l}\left(c\right)\right)$$

These enhancements make YOLOv9 a highly effective model for applications such as autonomous driving, traffic surveillance, vehicle recognition, and pedestrian detection.

#### 2.3.4. YOLOv8

YOLOv8 introduces further architectural improvements over its predecessors by refining the backbone and neck of the model [44]. This algorithm uses a CSPDarknet53 backbone for enhanced feature extraction and integrates a combination of feature pyramid networks and PANet in the neck, enabling the efficient aggregation of multiscale features. This structure enhances detection across varying object sizes and scales, making YOLOv8 particularly effective in real-time scenarios where high accuracy and speed are required. The YOLOv8 loss function comprises specific loss components designed to address different aspects of object detection: localization, classification, and bounding box refinement. Accordingly, focal loss (classification loss) is used and is calculated for each object instance, as shown in Equation (17).

(17) $$FL\left(p_{t}\right)=-\alpha \cdot {\left(1-p_{t}\right)}^{\gamma }\cdot log\left(p_{t}\right),$$

where $p_{t}$ is the model’s estimated probability for the true class, α is a balancing factor, and γ is a modulating factor to focus on hard, misclassified examples.

Distribution focal loss (DFL) is used for bounding box refinement. This function helps smooth bounding box predictions by leveraging a discretized distribution of regression targets, as shown in Equation (18).

(18) $$DFL=\sum_{k=0}^{reg\_max}w_{k}\cdot CE\left(p_{k},t_{k}\right),$$

where CE is the cross-entropy loss between the predicted distribution $p_{k}$ and the target $t_{k}$, and $w_{k}$ is a weight factor based on the bounding box offset distance. For object bounding boxes, the IoU (intersection over union) loss is computed as shown in Equation (19).

(19) $$IoU\;Loss-1-\frac{Intersection}{Union}$$

Additionally, CloU (complete IoU) is often used for precise localization, as shown in Equation (20).

(20) $$CIoU=IoU-\frac{\rho^{2}\left(b,b_{gt}\right)}{c^{2}}-\alpha v,$$

where ρ is the Euclidean distance between the centers of predicted and ground truth boxes, c is the diagonal length of the smallest enclosing box, and α and v account for the aspect ratio. The total loss function for YOLOv8 is expressed as shown in Equation (21).

(21) $$L_{total}=\lambda_{1}L_{box}+\lambda_{2}L_{obj}+\lambda_{3}L_{cls},$$

where $\lambda_{1},\lambda_{2}$, and $\lambda_{3}$ act as balancing weights. These enhancements increase the efficiency of YOLOv8 for real-time object detection, providing accuracy and speed.

#### 2.3.5. YOLOV5

YOLOv5 is an efficient single-stage object detection model that processes input images through its backbone, neck, and head, enabling fast and accurate detection [45]. The backbone uses cross-stage partial networks (CSPNet) to improve feature extraction while reducing computational workload. The neck is based on a path aggregation network, which integrates multiscale feature maps to improve detection across varying object sizes. The head generates the final predictions, including bounding box coordinates, objectness scores, and class probabilities. Optimization is conducted at three different scales to accommodate objects of various sizes. A key structural improvement in YOLOv5 lies in its balanced tradeoff between detection accuracy and computational speed. The model uses confidence scores and the three kinds of box regression-based classification losses in its loss function while measuring the difference between predicted and ground truth values. The loss function of the YOLOv5 model can be expressed as shown in Equation (22).

(22) $$L_{GIOU}=\sum_{i=0}^{s^{2}}\sum_{j=0}^{B}I_{ij}^{obj}\left[1-IOU+\frac{A^{c}-U}{A^{c}}\right],$$

where $S^{2}$ represents the number of grids, and B is the number of bounding boxes in each grid. The value of $I_{tj}^{obj}$ is equal to 1 in the presence of an object in the bounding box; otherwise, it will be 0.

(23) $$L_{conf}=-\sum_{i=0}^{s^{2}}\sum_{j=0}^{B}I_{ij}^{obj}\left[\overset{⊖}{C_{j}^{i}}\log\left(C_{i}^{j}\right)+\left(1-{\overset{⊖}{C}}_{i}\right)\log\left(1-C_{i}^{j}\right)\right]$$

(24) $$-\lambda_{noobj}\sum_{i=0}^{s^{2}}\sum_{j=0}^{B}I_{ij}^{noobj}\left[\overset{↷}{C_{i}}\log\left(C_{i}^{j}\right)+\left(1-C_{i}^{j}\right)\log\left(1-C_{i}^{j}\right)\right]$$

where ${\overset{C}{C}}_{t}$ is the predicted confidence of the bounding box of j in the grid of $i,C_{t}^{J}$ is the true confidence of the bounding box of j in the grid of i, and $\lambda_{noobj}$ is the confidence weight in the absence of objects in the bounding box.

(25) $$L_{class}=-\sum_{i=0}^{s^{2}}I_{ij}^{noobj}$$

(26) $$\sum_{c\in classes}\left[\overset{↷}{P_{i}^{j}}\left(c\right)\log\left(P_{i}^{j}\left(c\right)\right)+\left(1-\overset{P}{{\overbrace{P}}_{i}^{j}}\left(c\right)\right)\log\left(1-P_{i}^{j}\left(c\right)\right)\right],$$

where ${\overset{⇀}{P}}_{t}\left(c\right)$ is the probability that the detected object is predicted to belong to the category, and $P_{t}^{\prime }$ (c) is the probability that it actually belongs to the category.

## 3. Results

In the pipeline, each raw RGB skin lesion image was first processed by the SAVE module, which applied a learned regression mapping to convert the three-channel RGB input into a five-band narrowband representation. Its RGB counterpart was then uniformly resized to 640 × 640 pixels and normalized on a per-channel basis. Weight initialization was performed by duplicating pretrained RGB kernels where appropriate. During training, the combined loss (bounding box regression + object confidence + classification) was optimized using a learning rate of 0.005, a batch size of 16, and early stopping based on the validation F1 score. This integration enabled the YOLO model to leverage the enhanced spectral contrast provided by SAVE, resulting in improved lesion detection performance.

The performances of several YOLO object detection algorithms were evaluated for skin lesion detection, comparing their effectiveness under WLI and SAVE imaging modalities. The key performance metrics, which included precision, recall, and F1 scores, were assessed across four lesion categories: acrochordon, dermatofibroma, and lichenoid, as shown in Table 2.

**Figure 2:**
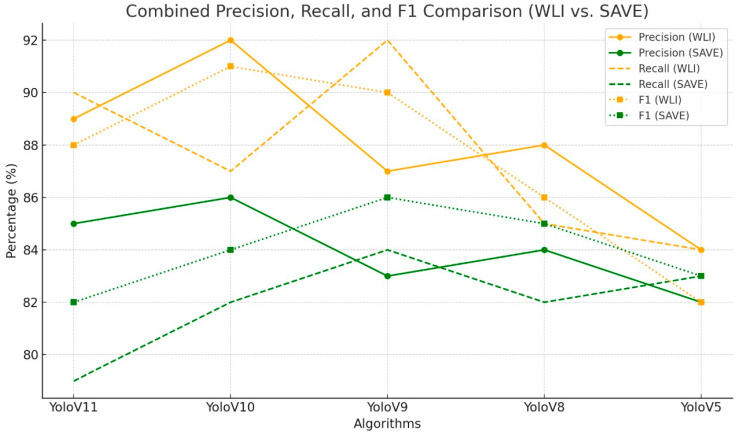
Comparative analysis of precision, recall, and F1 score for the detection of dermatofibroma using different object detection algorithms (YoloV11, YoloV10, YoloV9, YoloV8, and YoloV5) across two imaging modalities, including WLI and SAVE, for validation results.

The validation showed that SAVE outperformed WLI in object detection using YOLOv11, especially for classes such as acrochordon and lichenoid as shown in Figure 2. For lesions such as dermatofibroma, WLI demonstrated good detection capability, which was further enhanced by the additional spectral detail provided by SAVE. This improvement across all classes indicates that SAVE provides substantial contributions to the reliability of SC detection with YOLOv11 (Supplement Figure S1 for the loss and precision of WLI and SAVE of YoloV11; Figure S2 for the confusion matrix of WLI and SAVE of YoloV11; and Figure S3 for the F1–confidence curve of WLI and SAVE of YoloV11).

In the case of YOLOv10, SAVE achieved better validation results, particularly for lichenoid detection, which showed moderate performances with WLI but improved accuracy with SAVE. The overall consistency of the model increased with SAVE, especially for classes requiring high spectral contrast. These findings demonstrate that SAVE helps YOLOv10 in producing more accurate detections for lesion types that are typically difficult to identify compared to WLI (Supplement Figure S4 for the loss and precision of WLI and SAVE of YoloV10; Figure S5 for the confusion matrix of WLI and SAVE of YoloV10; and Figure S6 for the F1–confidence curve of WLI and SAVE of YoloV10).

The validation results of YOLOv9 demonstrated improved performance with SAVE, especially for complex classes. While WLI performed well for the dermatofibroma class, SAVE provided more accurate detection for the acrochordon and lichenoid classes. The positive results highlight the capability of SAVE to enhance spectral resolution and contrast, enabling YOLOv9 to effectively differentiate between small features in classes that are often difficult to identify under WLI (Supplement Figure S7 for the loss and precision of WLI and SAVE of YoloV9; Figure S8 for the confusion matrix of WLI and SAVE of YoloV9; and Figure S9 for the F1–confidence curve of WLI and SAVE of YoloV9).

YOLOv8 showed improved validation performance on classes such as acrochordon and lichenoid when used with SAVE. In contrast, WLI produced good results for well-defined classes. By highlighting the capability of YOLOv8 to identify key lesion characteristics, SAVE further improved the detection performance of the model. This finding reflects a consistent pattern across models, with SAVE substantially improving object detection accuracy, particularly for lesion types requiring remarkable spectral clarity (Supplement Figure S10 for the loss and precision of WLI and SAVE of YoloV8; Figure S11 for the confusion matrix of WLI and SAVE of YoloV8; and Figure S12 for the F1–confidence curve of WLI and SAVE of YoloV8).

Although YOLOv5 maintained balanced detection accuracy with WLI, especially for distinct classes such as dermatofibroma, SAVE contributed to improvements across all classes. During validation, SAVE enhanced the detection accuracy of YOLOv5, leading to highly accurate differentiation between classes. These findings support the idea that SAVE enhances the reliability of object detection (Supplement Figure S13 for the loss and precision of WLI and SAVE of YoloV5; Figure S14 for the confusion matrix of WLI and SAVE of YoloV5; and Figure S15 for the F1–confidence curve of WLI and SAVE of YoloV5).

Validation results across all YOLO models demonstrated that SAVE consistently outperformed WLI in the detection of SC. By enhancing spectral information and visual contrast, SAVE proved particularly effective for complex lesion types, such as lichenoid and acrochordon. Based on these results, integrating SAVE into the detection workflow improves detection reliability. Thus, SAVE is a useful technique for improving performance in medical imaging tasks using object detection models such as YOLO (Supplement Figure S16 for the comparison of WLI and SAVE results, highlighting the contrast between the normal and the SAVE images).

This study evaluated the performance of five YOLO models—YOLOv11, YOLOv10, YOLOv9, YOLOv8, and YOLOv5—across two imaging modalities, WLI and SAVE. The models were tested on the following three lesion classes: dermatofibroma, acrochordon, and lichenoid. The evaluation metrics considered included precision, recall, and F1 score. YOLOv10 demonstrated the highest accuracy, particularly in classifying all lesion types using the SAVE modality, where precision and recall were notably higher compared to WLI, as shown in Table 3. Analysis of SAVE results showed that the F1 scores for acrochordon and dermatofibroma were high, indicating the effectiveness of the model in improving precision and recall. This finding also implies that YOLOv10 is highly accurate in cancer detection within the SAVE modality, outperforming the other models. In the case of YOLOv9, the performance metrics were slightly lower than those of YOLOv10, but it still produced strong precision and recall values across all classes in the WLI modality. However, the model achieved strong performance under the SAVE modality, particularly for dermatofibroma. YOLOv9 demonstrated good generalization across both modalities, although its results were slightly lower than those of YOLOv10, especially in WLI for acrochordon detection. YOLOv11 achieved an overall F1 score of 65.73%, indicating good performance across all classes, particularly with the SAVE method. However, its accuracy in detecting acrochordon was lower compared to dermatofibroma and lichenoid. Overall, better performance was observed with the SAVE modality, where overall precision, recall, and F1 scores were higher than in WLI, especially for acrochordon, which demonstrated weaker results under WLI. These results indicate that, despite the excellent performance of YOLOv11, it does not surpass YOLOv10 in terms of precision and recall metrics. Among all the models, YOLOv8 had the lowest accuracy and F1 score, particularly on the WLI modality. This finding indicates that YOLOv8 is least effective in detecting some classes, particularly acrochordon. Although the SAVE modality improved the performance of YOLOv8, its results were still lower compared to those of YOLOv9 and YOLOv10. Thus, YOLOv8 was less precise with WLI and produced more false positives than the newer versions of YOLO. YoloV5, despite being from an earlier model generation, performed well in both imaging modalities, particularly when using the SAVE method. Its performance was only slightly lower than that of YOLOv9 and YOLOv10. Although the results showed lower precision compared to the latest YOLO models, YOLOv5 remained an effective model for lesion diagnosis, especially with the SAVE modality. After comparing all the results from YOLOv11, YOLOv8, and YOLOv5, notably higher precision was observed in YOLOv10 and YOLOv9, particularly with the SAVE modality, where both models performed substantially better. This finding indicates that the SAVE imaging method provides high accuracy. Considering the evaluation metrics, YOLOv10 achieved the highest precision and recall values, making it the best-performing algorithm overall. In contrast, YOLOv11 could have performed better in WLI and did not surpass the other models, especially when using SAVE. Overall, improvements in SC detection performance were greatest for the YOLOv10 and YOLOv9 models when using the SAVE imaging modality.

**Figure 3:**
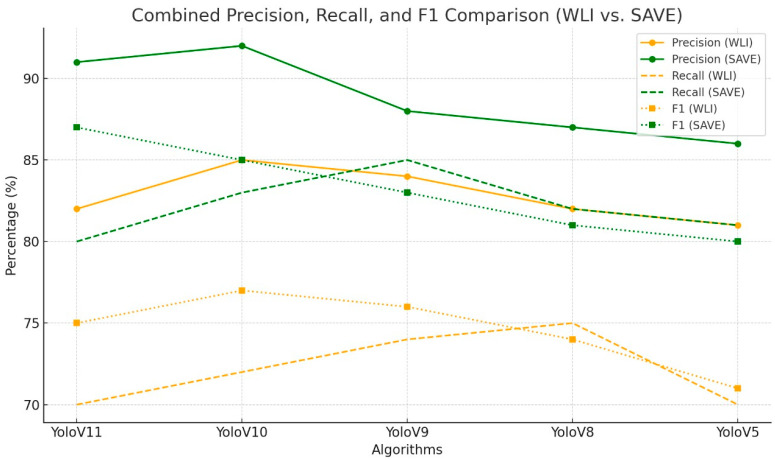
Comparative analysis of precision, recall, and F1 score for the detection of dermatofibromas using different object detection algorithms (YoloV11, YoloV10, YoloV9, YoloV8, and YoloV5) across two imaging modalities, which included WLI and SAVE, for testing results.

**Figure 4:**
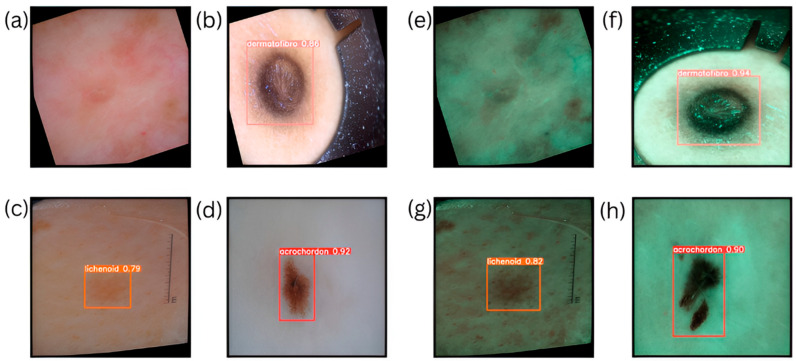
Detection results: WLI—(**a**) represents the normal class, (**b**) represents the bounded box with dermatofibroma, (**c**) represents the bounded box with lichenoid, and (**d**) represents the bounded box with acrochordon. SAVE—(**e**) represents the normal class, (**f**) represents the bounded box with dermatofibroma, (**g**) represents the bounded box with lichenoid, and (**h**) represents the bounded box with acrochordon.

Acrochordon emerged as the most challenging lesion category across all models and imaging techniques, consistently yielding the lowest F1 scores, even after SAVE conversion as shown in Figure 3 (ranging from 49.69% for YOLOv11–SAVE to 66.60% for YOLOv8–SAVE). Multiple factors possibly contributed to this difficulty, including the small size of the lesions—skin tags typically occupy only a few dozen pixels, even after resizing to 640 × 640, rendering them challenging to localize and distinguish from background noise. Additionally, acrochordons exhibit considerable variations in color, texture, and attachment morphology, reducing the effectiveness of RGB and narrowband contrast cues as shown in Figure 4. In contrast, dermatofibromas display distinct vascular patterns that are effectively captured by the spectral bands of SAVE. Although SAVE enhanced recall (for instance, YOLOv10’s recall for acrochordon increased from 42.9% to 49.7%), improvements in precision were highly modest, indicating persistent false positives involving other minor skin features. Aiming to address these challenges, future efforts should enhance the acrochordon subset through targeted oversampling and synthetic data creation using controlled cropping and color jittering to accentuate tag-like textures. Additionally, exploring alternative narrowband wavelengths may improve the discrimination of skin tag structures. Customized strategies will be essential to improve acrochordon detection performance to levels comparable with more easily recognizable lesions.

## 4. Discussion

SAVE consistently produced statistically significant improvements in F1 across all models, with YOLOv10 showing a notable increase of +10.3 percentage points. This finding highlights the effectiveness of snapshot-based narrowband contrast in enhancing detection performance. Unlike device-specific filters such as i-Scan or FICE, a regression-based framework attains comparable or superior lesion detection efficacy without requiring specialized hardware. These findings indicate that SAVE not only connects RGB and hyperspectral methodologies but also outperforms current pseudo-HSI techniques in practical detection applications. The results from this study indicate promising advancements in SC diagnosis; however, several limitations must be addressed to enable further improvements. First, the computational load associated with HSI, machine learning algorithms, and video analysis remains a major challenge for scaling these technologies to real-world applications [46]. Advanced computing resources such as GPUs, TPUs, and FPGAs are essential to reduce processing time and allow real-time diagnostics [47]. Moreover, the use of ensemble models combined with transfer learning enhances system performance by leveraging pretrained networks, thus avoiding the need for exhaustive training on large new datasets and improving model accuracy and reliability [48].

### 4.1. Uncertainty Analysis

A major limitation to consider relates to the dataset. This study was conducted using data from an open-source resource, the ISIC platform, which has broad demographic coverage. However, incorporating additional data from different clinical and geographical settings would enhance the generalizability of this model. Although the ISIC archive offers a valuable and meticulously annotated benchmark, dependence on a single public dataset may inadequately reflect the variability encountered in clinical practice, such as differences in imaging devices, lighting conditions, skin phototypes, and actual lesion presentations. Consequently, the performance of the model may vary when applied to smartphone-acquired images, cross-institutional dermoscopic systems, or patient demographics exhibiting a range of skin tones. Future work will include prospective validation on multicenter clinical cohorts, refinement and domain adaptation for smartphone-acquired images and underrepresented skin types, and a pilot implementation in dermatology clinics to assess real-time detection accuracy and workflow integration. Such expansion will improve the model’s adaptability and ensure broader applicability across diverse patient populations.

### 4.2. Limitations and Computational Cost

Another limitation involves the preprocessing of images. In this study, all images were resized to a standard resolution of 640 pixels to reduce computational complexity and enhance data consistency [49]. However, this resizing may result in the loss of critical details for SC analysis. Future research should investigate adaptive resolution techniques that preserve high-resolution features where necessary, balancing image quality with computational efficiency. With frame-by-frame processing, optimizing the quality and computational speed of video analysis may be possible by capturing minimal changes that static images may fail to capture. Despite its robust detection capabilities, the proposed methodology possesses two primary limitations: computational resources and algorithmic scope. Aiming to obtain more robust and less optimistic performance estimates, future studies should implement stratified k-fold or nested cross-validation across RGB and SAVE datasets. Distributed training frameworks will also be essential to manage the increased computational load. Additionally, a systematic ablation study will be conducted to evaluate the impact of individual components, such as SAVE’s spectral band selection, normalization pipeline, and network architecture, on detection performance. The current study was based primarily on static, visual-based SC identification; extending the data analysis to video formats could introduce novel possibilities for improving diagnostic accuracy. By tracking lesion variations over time, video-based analysis could help identify dynamic patterns indicative of disease progression, which may be particularly valuable in early cancer detection. Subsequent research will also examine the application of novel architectures such as SimPoolFormer, FDSSC, Tri-CNN, and other CNN models to further improve SC detection using SAVE-enhanced imaging [50,51,52].

### 4.3. Comparison to Alternative Spectral Modalities and Clinical Translation

MSI and HSI systems provide intricate spatiospectral tissue signatures; however, these systems depend on cumbersome scanning apparatuses or costly tunable filters, limiting their regular clinical implementation. In endoscopy, solutions such as Pentax i-Scan and Fujinon FICE use proprietary lookup tables on RGB sensors. However, they provide limited transparency and necessitate vendor-specific platforms. NBI is extensively validated and grounded in well-defined hemoglobin absorption peaks; however, it generally necessitates modified light sources. In contrast, SAVE holds a distinctive role: it emulates the vascular contrast of NBI through a learned regression model applied to standard RGB data. Thus, SAVE is entirely hardware-agnostic, capable of generating five-band narrowband outputs from a single image capture using conventional equipment. In addition to promoting early action, SAVE has the potential to improve patient outcomes by enabling thorough and dynamic examinations. The extent of diagnosis and optimization for clinical usage should receive increased attention in future research. Further development for static images and real-time video data is essential to achieving an accurate, responsive evaluation system that enhances diagnostic precisions and clinical decision-making. As this technology matures, this approach is expected to become a vital tool in the medical field.

## 5. Conclusions

Skin disorders such as acrochordon, dermatofibroma, and lichenoids can be effectively identified by incorporating HSIs with advanced object detection algorithms, such as Yolov11, YOLOv10, YOLOv9, YOLOv8, and YOLOv5. While all models demonstrated excellent performance, YOLOv10 achieved the best overall results across key evaluations metrics—accuracy, precision, recall, and F1 score—particularly for the dermatofibroma class. YOLOv9 and YOLOv8 also performed well, confirming the effectiveness of modern YOLO architectures in skin lesion detection. This study indicates that deploying these advanced YOLO models in conjunction with HSI can notably enhance the accuracy and efficiency of skin lesion detection. These methods hold strong potential for clinical applications, especially in cases where early diagnosis is critical to successful treatment outcomes. The integration of real-time object detection into dermatological imaging systems could assist clinicians in identifying skin abnormalities with remarkable speed and precision. Further studies should focus on finetuning these models with highly diverse patient datasets and exploring the application of HSI combined with YOLO models in diagnosing other types of skin cancers or skin conditions. Such advancements could enable timely diagnosis, leading to better-targeted treatment methods and ultimately improving patient survival rates when timely intervention is needed.

## Figures and Tables

**Table 1:** Dataset used in this study.

Lesion Type	Total Images	Training (70%)	Validation (20%)	Test (10%)
Acrochordon	577	404 (70%)	116 (20%)	57 (10%)
Dermatofibroma	821	575 (70%)	164 (20%)	82 (10%)
Lichenoid	805	563 (70%)	161 (20%)	81 (10%)
Total	2203	1542 (70%)	441 (20%)	220 (10%)

**Table 2:** Validation results of multiple models.

Algorithm	Imaging Modality	Class	Precision in %	Recall in %	F1 in %
YoloV11	WLI	All	71	62.9	66.71
		Acrochordon	62.8	40	48.87
		Dermatofibroma	82.3	89.7	85.84
		Lichenoid	67.9	59	63.14
	SAVE	All	74.3	54.2	62.68
		Acrochordon	70.9	39.8	50.98
		Dermatofibroma	86.1	76.3	80.9
		Lichenoid	65.8	46.5	54.49
YoloV10	WLI	All	79.2	58.6	67.36
		Acrochordon	69.3	34.2	45.8
		Dermatofibroma	90.9	83.3	86.93
		Lichenoid	77.4	58.2	66.44
	SAVE	All	79.2	58.3	67.16
		Acrochordon	72.3	39.2	50.84
		Dermatofibroma	90.1	81.4	85.53
		Lichenoid	75.1	54.4	63.1
YoloV9	WLI	All	75.8	67	71.13
		Acrochordon	62.8	48.8	54.92
		Dermatofibroma	87.9	90.4	89.13
		Lichenoid	76.8	61.9	68.55
	SAVE	All	86.4	56.1	68.03
		Acrochordon	83.6	36.7	51.01
		Dermatofibroma	87.8	79.2	83.28
		Lichenoid	87.9	52.6	65.82
YoloV8	WLI	All	78.2	60.8	68.41
		Acrochordon	64.4	32.8	43.46
		Dermatofibroma	89.5	87.5	88.49
		Lichenoid	80.8	61.9	70.1
	SAVE	All	81.2	57.9	67.6
		Acrochordon	74.8	36.7	49.24
		Dermatofibroma	88.2	79.9	83.85
		Lichenoid	88.5	57.2	69.49
YoloV5	WLI	All	70.7	62.8	66.52
		Acrochordon	57.8	40.5	47.63
		Dermatofibroma	85.1	84.3	84.7
		Lichenoid	69.1	63.4	66.13
	SAVE	All	71.6	59.3	64.87
		Acrochordon	64.4	39.2	48.74
		Dermatofibroma	81.8	81.5	81.65
		Lichenoid	68.5	57.2	62.34

**Table 3:** Testing results of multiple machine learning models.

Algorithm	Imaging Modality	Class	Precision in %	Recall in %	F1 in %
YoloV11	WLI	All	74.5	50.7	57.67
		Acrochordon	72.6	37.9	49.91
		Dermatofibroma	83.7	66.7	73.77
		Lichenoid	67.2	47.4	55.71
	SAVE	All	77.5	57.9	65.73
		Acrochordon	58.1	43.3	49.69
		Dermatofibroma	93.4	82.7	87.04
		Lichenoid	80.9	47.6	60.17
YoloV10	WLI	All	70	52.9	60.3
		Acrochordon	56.4	42.9	48.7
		Dermatofibroma	85.4	68.5	76
		Lichenoid	68	47.4	55.9
	SAVE	All	85	60.3	70.6
		Acrochordon	75.7	49.7	60
		Dermatofibroma	92	79.3	85.2
		Lichenoid	87.3	52.1	65.3
YoloV9	WLI	All	81.4	56.2	66.5
		Acrochordon	85	47.6	61
		Dermatofibroma	85	70.8	77.3
		Lichenoid	74.1	50.2	59.9
	SAVE	All	87.3	60.6	71.5
		Acrochordon	79.1	50	61.3
		Dermatofibroma	92.3	81.7	86.7
		Lichenoid	90.3	50	64.4
YoloV8	WLI	All	80.4	55.9	65.9
		Acrochordon	78.8	45.2	57.4
		Dermatofibroma	87.5	71.9	78.9
		Lichenoid	74.9	50.5	60.3
	SAVE	All	83.3	64.3	72.6
		Acrochordon	80.5	56.8	66.6
		Dermatofibroma	88.4	84.1	86.2
		Lichenoid	80.9	52.1	63.4
YoloV5	WLI	All	68	49.5	57.3
		Acrochordon	60.8	33.3	43
		Dermatofibroma	80.9	67.4	73.5
		Lichenoid	62.3	47.8	54.1
	SAVE	All	74.7	61.4	67.4
		Acrochordon	63.2	52.3	57.2
		Dermatofibroma	87.5	82.9	85.1
		Lichenoid	73.4	49	58.8

## Data Availability

The data presented in this study are available in this article upon request to the corresponding author. The data are not publicly available due to participant privacy and confidentiality restrictions; they contain sensitive personal health information that cannot be openly shared.

## Supplementary Materials

The following supporting information can be downloaded at: https://www.mdpi.com/article/10.3390/bioengineering12070714/s1, Figure S1 represents the loss and precision of WLI and SAVE; Figure S2 represents the confusion matrix of WLI and SAVE; Figure S3 represent the F1–confidence curve of WLI and SAVE; Figure S4 represents the loss and precision of WLI and SAVE; Figure S5 represents the confusion matrix of WLI and SAVE; Figure S6 represent the F1–confidence curve of WLI and SAVE; Figure S7 represents the loss and precision of WLI and SAVE; Figure S8 represents the confusion matrix of WLI and SAVE; Figure S9 represent the F1–confidence curve of WLI and SAVE; Figure S10 represents the loss and precision of WLI and SAVE; Figure S11 represents the confusion matrix of WLI and SAVE; Figure S12 represent the F1–confidence curve of WLI and SAVE; Figure S13 represents the loss and precision of WLI and SAVE; Figure S14 represents the confusion matrix of WLI and SAVE; Figure S15 represent the F1–confidence curve of WLI and SAVE. Figure S16 Comparison of WLI and SAVE results highlighting the contrast between the normal and the SAVE images. Figure S17 Comparison of SSIM between the simulated NBI images and the WLI images of VCE and Olympus. Figure S18 Comparison of entropy between the simulated NBI images and the WLI images. (a) Entropy for Olympus Endoscopy while (b) shows the entropy for the VCE camera. Figure S19 Comparison of PSNR of the twenty randomly chosen images in Olympus and VCE. Figure S20 Endoscopic using three imaging techniques. (a) WLI, (b) NBI, (c) SAVE. Figure S21 The lighting spectrum difference between the Olympus WLI, Olympus NBI, and the Capsule WLI. Figure S22 The Difference between the Olympus SAVE and the VCE simulated NBI lighting. (a) shows the difference between the Olympus SAVE and Olympus WLI. (b) shows the difference between VCE SAVE and VCE WLI images. Table S1 SSIM of twenty randomly chosen images in VCE and Olympus endoscope. Table S2 Entropy comparison of the WLI and NBI images in Olympus and VCE endoscope. Table S3 Comparison of PSNR of the twenty randomly chosen images in Olympus and VCE. Table S4 Comparison of F1-score of the different models.
