# Supplementary material for: Novel Snapshot-Based Hyperspectral Conversion for Dermatological Lesion Detection via YOLO Object Detection Models
Source: Bioengineering (Basel). 2025 Jun 30;12(7):714. doi: 10.3390/bioengineering12070714 (PMC12292811; doi:10.3390/bioengineering12070714)
Supplement: Supplementary file 1 [file bioengineering-12-00714-s001.zip › bioengineering-3668844-supplementary.pdf]

bioengineering

MDPI

Article

Application of a Novel Spectral Enhancer for Skin Cancer Detection: Supplementary

Nan-Chieh Huang [1,2], Arvind Mukundan [3,4], Riya Karmakar [3], Syna Syna [5], Wen-Yen Chang [6,*], and Hsiang-Chen Wang [3,7,8,*]

1 Diving Medical and Physiology Training Center. Zuoying Armed Forces General Hospital, No. 553, Junxiao Rd., Zuoying Dist., Kaohsiung City 813204, Taiwan; pippen0050@gmail.com (N.-C.H.)
2 Department of Information Engineering, I-Shou University, No.1, Sec. 1, Syuecheng Rd., Dashu District, Kaohsiung City 84001, Taiwan.
3 Department of Mechanical Engineering, National Chung Cheng University, 168, University Rd., Min Hsiung, Chia Yi 62102, Taiwan; karmakarriya345@gmail.com (R.K.); arvindmkukund96@gmail.com (A.M.)
4 Department of Biomedical Imaging, Chennai Institute of Technology, Sarathy Nagar, Chennai 600069, India
5 Chitkara University, Department of Computer Science and Engineering, Chandigarh-Patiala National Highway (NH- 64 Village Jansla, Rajpura, Punjab 140401, India; synab6498@gmail.com
6 Department of General Surgery, Kaohsiung Armed Forces General Hospital, 2, Zhongzheng 1st. Rd., Kaohsiung City 80284, Taiwan; wenyen85@gmail.com (W.-Y.C.)
7 Department of Medical Research, Dalin Tzu Chi Hospital, Buddhist Tzu Chi Medical Foundation, No. 2, Minsheng Road, Dalin, Chiayi, 62247 Taiwan
8 Director of Technology Development, Hitspectra Intelligent Technology Co., Ltd., Kaohsiung 80661, Taiwan
* Correspondence: wenyen85@gmail.com (W.-Y.C.) and hcwang@ccu.edu.tw (H.-C.W.)

Academic Editor: Firstname Lastname

Received: date
Revised: date
Accepted: date
Published: date

Citation: To be added by editorial staff during production.

Copyright: © 2025 by the authors. Submitted for possible open access publication under the terms and conditions of the Creative Commons Attribution (CC BY) license (https://creativecommons.org/licenses/by/4.0/).

S1. Evaluation Indices

Evaluation metrics are mathematical, quantitative, and objective measures for grading the accuracy of performance of an efficacy of statistical or machine learning algorithms. These provide very important information with respect to understanding how the model is performing, help in the comparison of different models, or return that could be the same model with the same algorithm configuration. Accuracy measures the percentage of samples in the data set that were correctly categorized by the model as positive, thus measures the capacity of the model to avoid the false positives.

$$\text{Accuracy} = \frac{TP+TN}{TP+TN+FP+FN} \quad \text{(S1)}$$

Recall computes the ratio of correct positive cases relative to all the cases that are positive actually; it deals with the capacity of the model to diagnose all instances of a particular class.

$$\text{Precision} = \frac{tp}{tp+fp}$$
$$\text{Recall} = \frac{tp}{tp+fn} \quad \text{(S2)}$$

The F1 Score, which is formed by the precision score and the recall score, and summing them. The F-1 Score, therefore, delivers a more accurate account of the performance of the model in the balanced mode either of false positives or false negatives.

$$F = 2 \cdot \frac{\text{precision} \cdot \text{recall}}{\text{precision} + \text{recall}} \quad \text{(S3)}$$

Recall ranges from 0 to 1, and the calculation of the mean of average precisions was done. The mAP formula depends upon the following sub-metrics: Confusion matrix, Intersection over Union IoU, Miss rate, Hit rate. Four characteristics are required to develop the confusion matrix, which is True Positive, True Negatives, True Negative, and False Negatives. mAP50 is interpreted as the Mean average precision, calculated at the intersection over union of 0.50. It has something to do with how well the model performs based on the subset of detections that every algorithm should ideally be able to handle easily. mAP50-95 is the mean average precision at the intersection of union of different levels of

IoU thresholds from 0.50 to 0.95. Therefore, it gives an overview of the performance of the model with respect to object findability in an image.

$$\text{mAP} = \frac{1}{N}\sum_{i=1}^{N} \text{AP}_i \quad \text{(S4)}$$

S2. Spectrum-Aided Vision Enhancer

The discrete alteration formulas to adapt the 24-color patch image and 24 color patch reflectance spectrum data to XYZ color space are as follows

On the camera part: alter sRGB color gamut space to XYZ color gamut space

$$\begin{bmatrix} X \\ Y \\ Z \end{bmatrix} = [M_A][T]\begin{bmatrix} f(R_{sRGB}) \\ f(G_{sRGB}) \\ f(B_{sRGB}) \end{bmatrix} \times 100\,, 0 \le \begin{matrix} R_{sRGB} \\ G_{sRGB} \\ B_{sRGB} \end{matrix} \le 1 \quad \text{(S5)}$$

$$[T] = \begin{bmatrix} 0.4104\ 0.3576\ 0.1805 \\ 0.2126\ 0.7152\ 0.0722 \\ 0.0193\ 0.1192\ 0.9505 \end{bmatrix} \quad \text{(S-6)}$$

$$f(n) = \begin{cases} (\frac{n+0.055}{1.055})^{2.4}, n > 0.04045 \\ \left(\frac{n}{12.92}\right), otherwise \end{cases} \quad \text{(S-7)}$$

$$[M_A] = \begin{bmatrix} X_{SW}/X_{CW} & 0 & 0 \\ 0 & Y_{SW}/Y_{CW} & 0 \\ 0 & 0 & Z_{SW}/Z_{CW} \end{bmatrix} \quad \text{(S-8)}$$

$$Z = k\int_{400nm}^{700nm} S(\lambda)R(\lambda)\bar{z}(\lambda)d\lambda \quad \text{(S-9)}$$

$$k = 100/\int_{400nm}^{700nm} S(\lambda)\bar{y}(\lambda)d\lambda \quad \text{(S-10)}$$

The nonlinear response of the camera can be modified by a third-order formula, and the nonlinear response alteration variable is demarcated as $V_{\text{Non-linear}}$.

$$V_{Non-linear} = [X^3\ Y^3\ Z^3\ X^2\ Y^2\ Y^2\ X\ Y\ Z\ 1]^T \quad \text{(S-11)}$$

Within the dark current component of an imaging device, the dark current typically maintains a consistent value regardless of the quantity of light received. As such, a fixed value is assigned to represent the dark current's contribution, and the dark current modification variable is established accordingly VDark.

$$V_{Dark} = [a] \quad \text{(S-12)}$$

The variable matrix V is gotten by standardizing the product of VColor and VNon-linear, with the addition of VDark. To prevent over-correction, the standardization is limited to the third order.

$$V_{Color} = [XYZ\ XY\ XZ\ YZ\ X\ Y\ Z]^T \quad \text{(S-13)}$$

$$V = \begin{bmatrix} X^3\ Y^3\ Z^3 \\ X^2Y\ X^2Z\ Y^2Z \\ XY^2\ XZ^2\ YZ^2 \\ XYZ\ X^2\ Y^2\ Y^2 \\ XY\ XZ\ YZ\ X\ Y\ Z\ a \end{bmatrix}^T \quad \text{(S-14)}$$

Prior to utilizing CIE DE2000 for color difference computation, it is necessary to convert XYZCorrect and XYZSpectrum from the XYZ color space to the lab color space. The equation for conversion is as ensues:

$$\begin{aligned} L^* &= 116f\left(\frac{Y}{Y_n}\right) - 16 \\ a^* &= 500\left[f\left(\frac{X}{X_n}\right) - f\left(\frac{Y}{Y_n}\right)\right] \\ b^* &= 200\left[f\left(\frac{Y}{Y_n}\right) - f\left(\frac{Z}{Z_n}\right)\right] \end{aligned} \quad \text{(S-15)}$$

$$f(n) = \begin{cases} n^{\frac{1}{3}},\ n > 0.008856 \\ 7.787n + 0.137931, otherwise \end{cases} \quad \text{(S-16)}$$

S3. Results

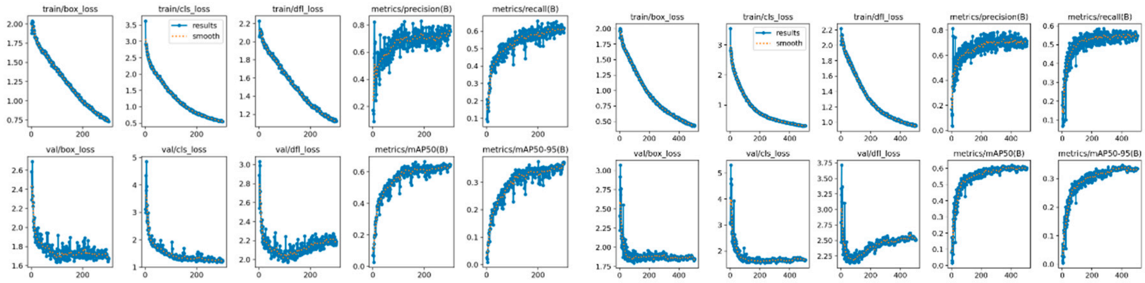

Figure S1 represents the loss and Precision of WLI and SAVE of YoloV11

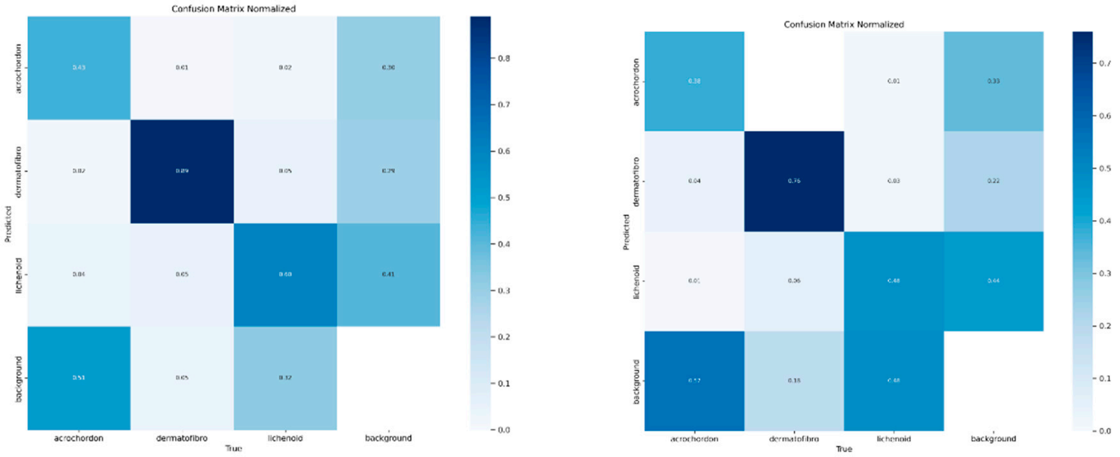

Figure S2 represents the confusion matrix of WLI and SAVE of YoloV11

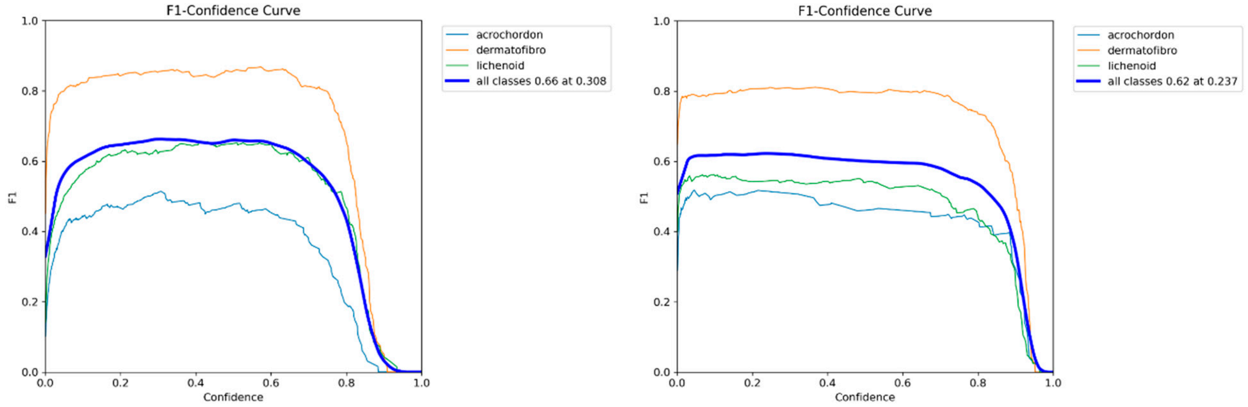

Figure S3 represent the F1-Confidence curve of WLI and SAVE of YoloV11

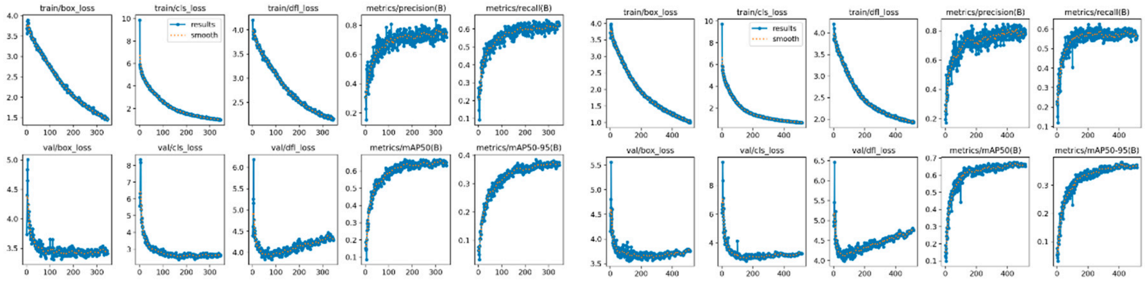

Figure S4 represents the loss and Precision of WLI and SAVE of YoloV10

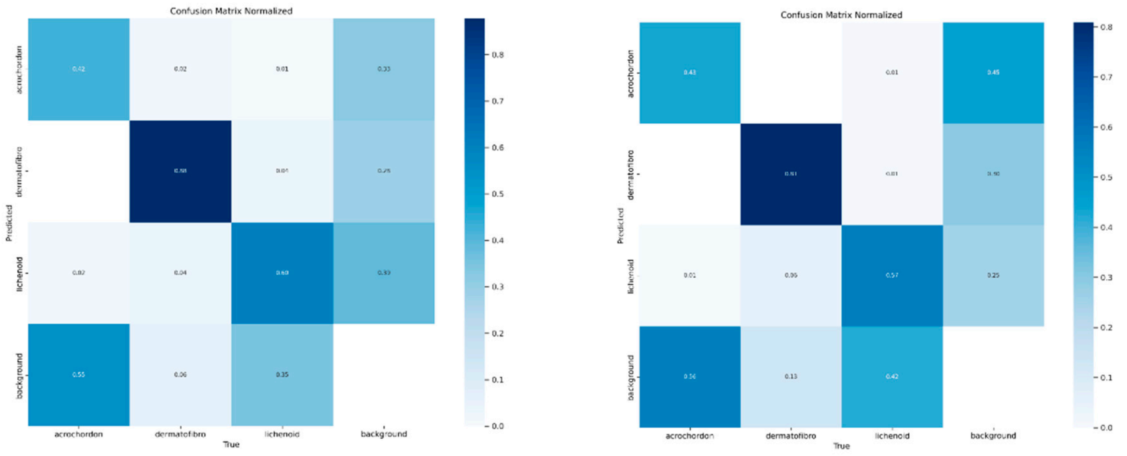

Figure S5 represents the confusion matrix of WLI and SAVE of YoloV10

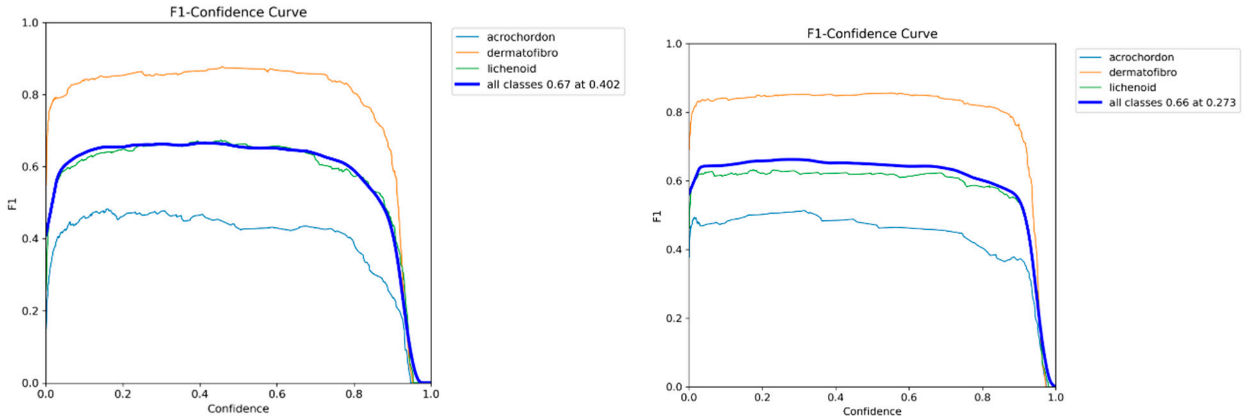

Figure S6 represent the F1-Confidence curve of WLI and SAVE of YoloV10

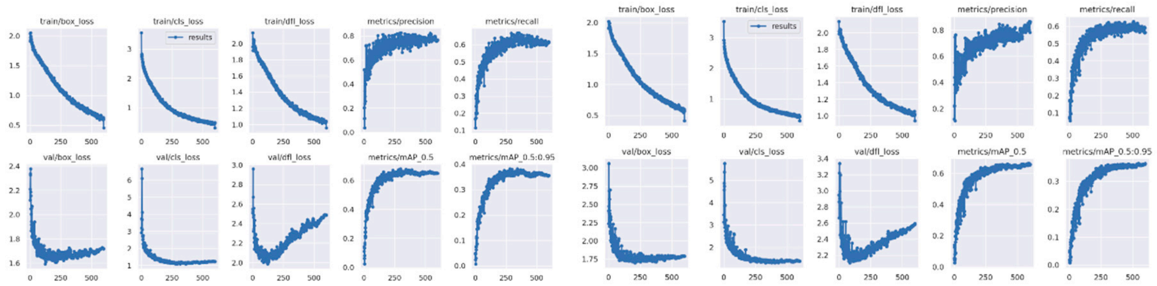

Figure S7 represents the loss and Precision of WLI and SAVE of YoloV9

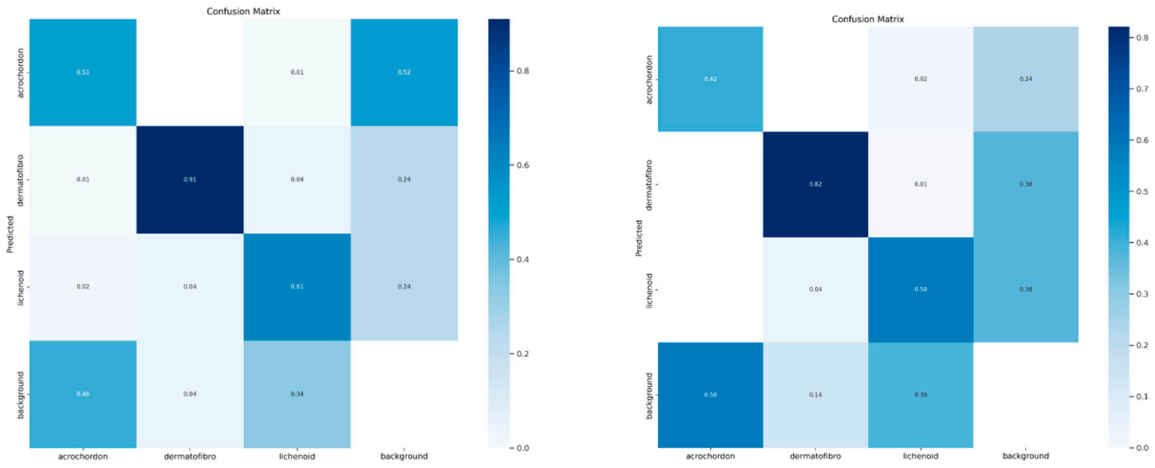

Figure S8 represents the confusion matrix of WLI and SAVE of YoloV9

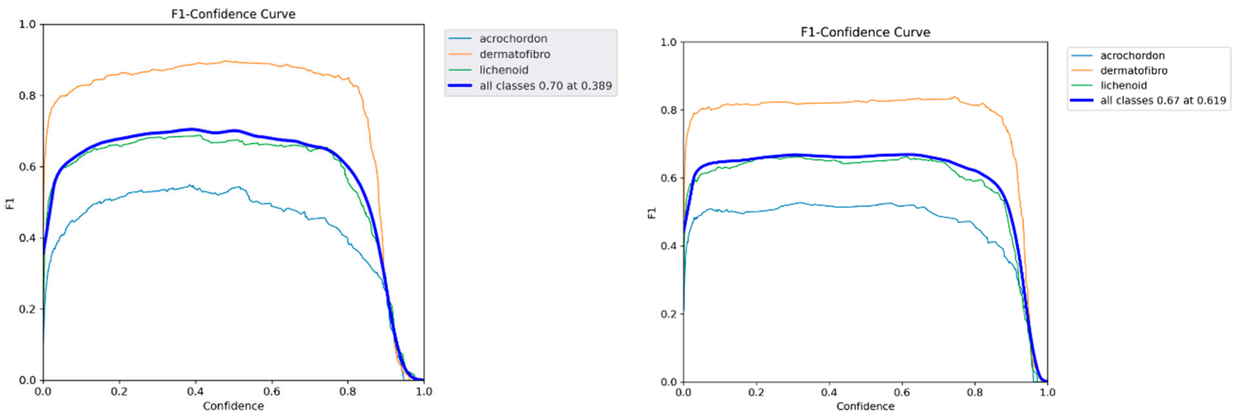

Figure S9 represent the F1-Confidence curve of WLI and SAVE of YoloV9

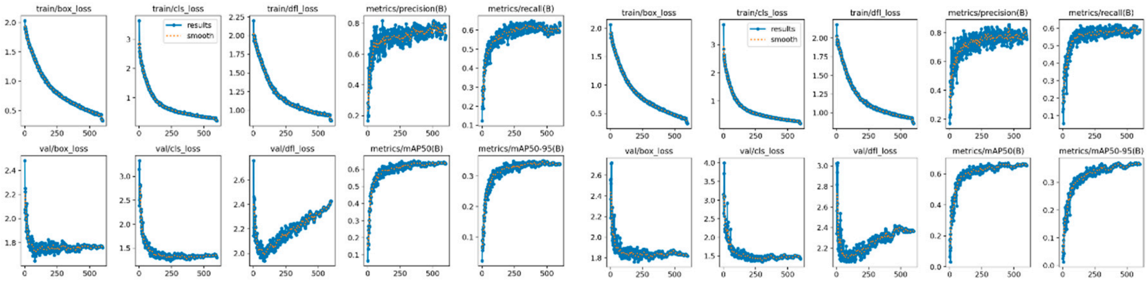

Figure S10 represents the loss and Precision of WLI and SAVE of YoloV8

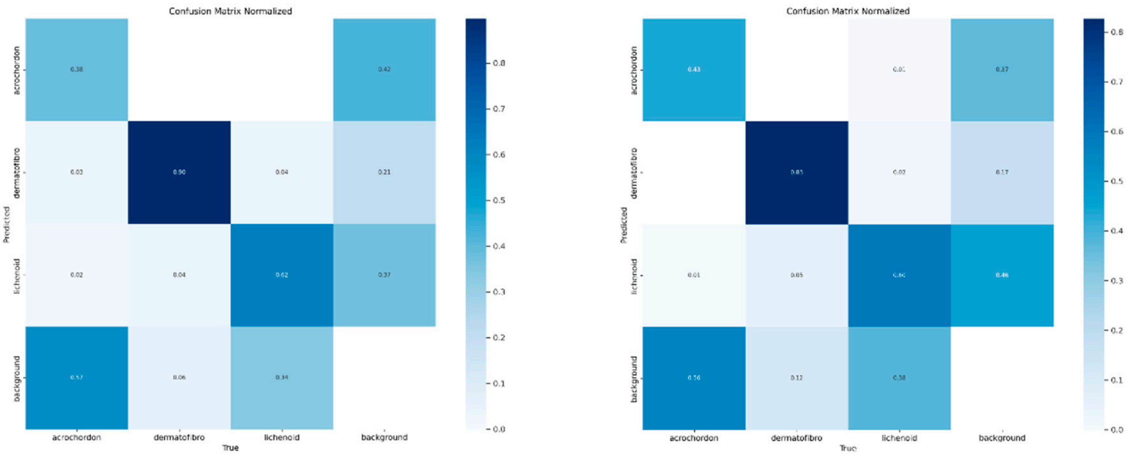

Figure S11 represents the confusion matrix of WLI and SAVE of YoloV8

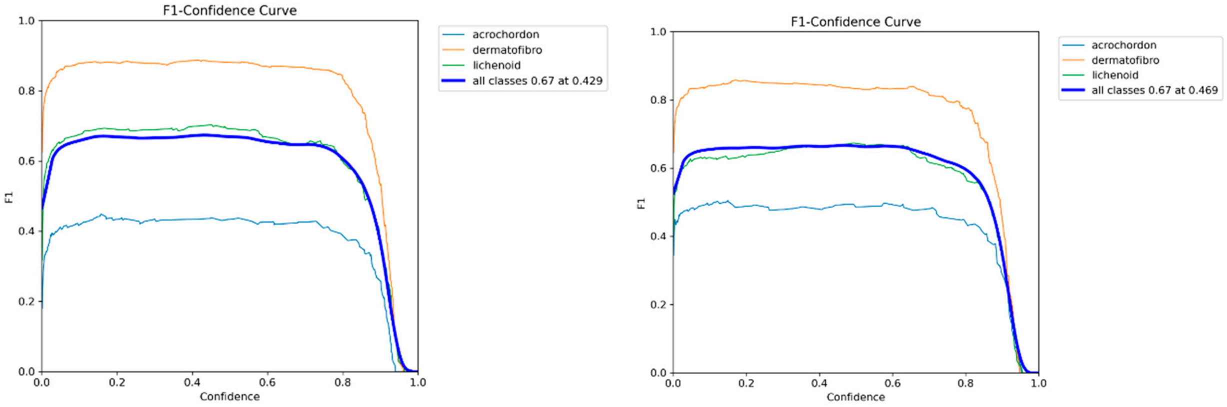

Figure S12 represent the F1-Confidence curve of WLI and SAVE of YoloV8

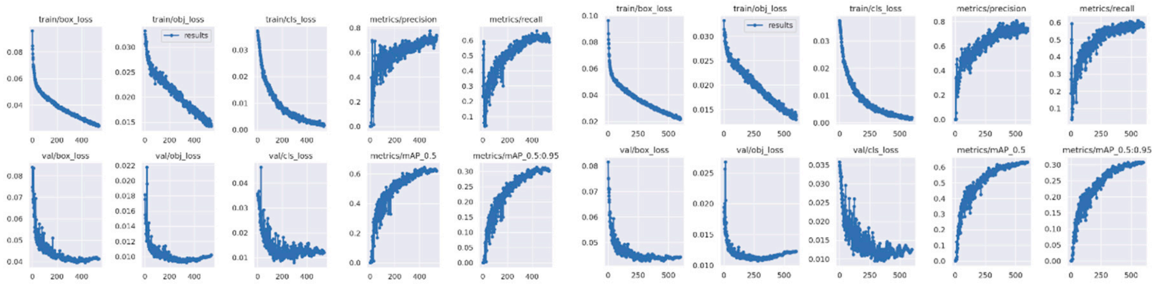

Figure S13 represents the loss and Precision of WLI and SAVE of YoloV5

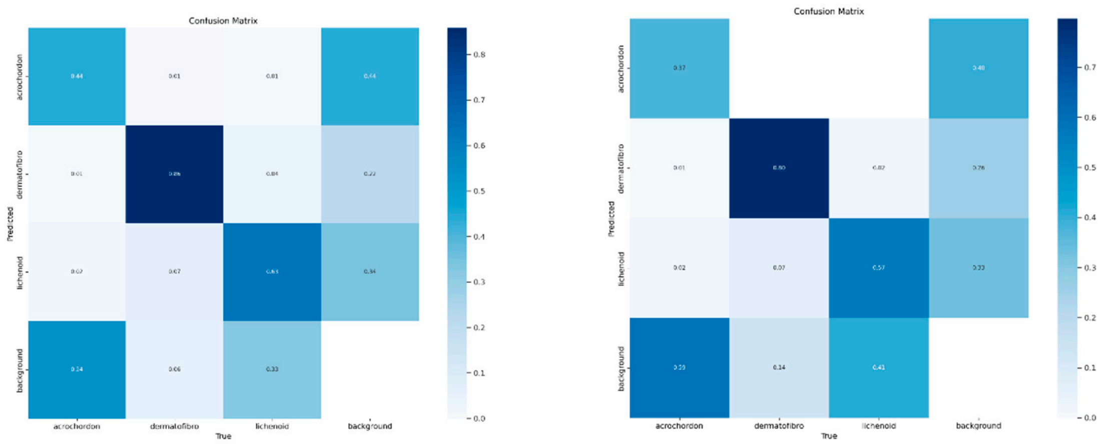

Figure S14 represents the confusion matrix of WLI and SAVE of YoloV5

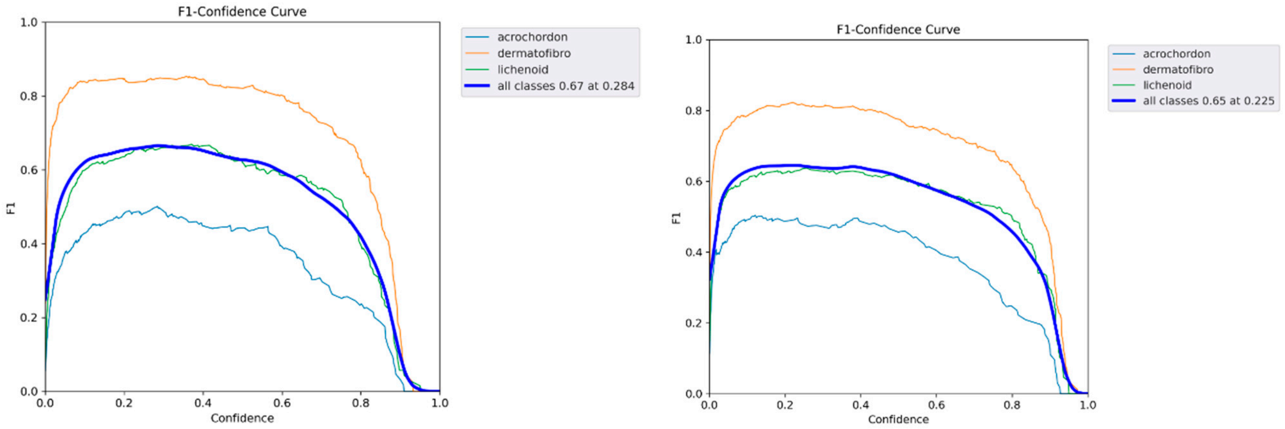

Figure S15 represent the F1-Confidence curve of WLI and SAVE of YoloV5

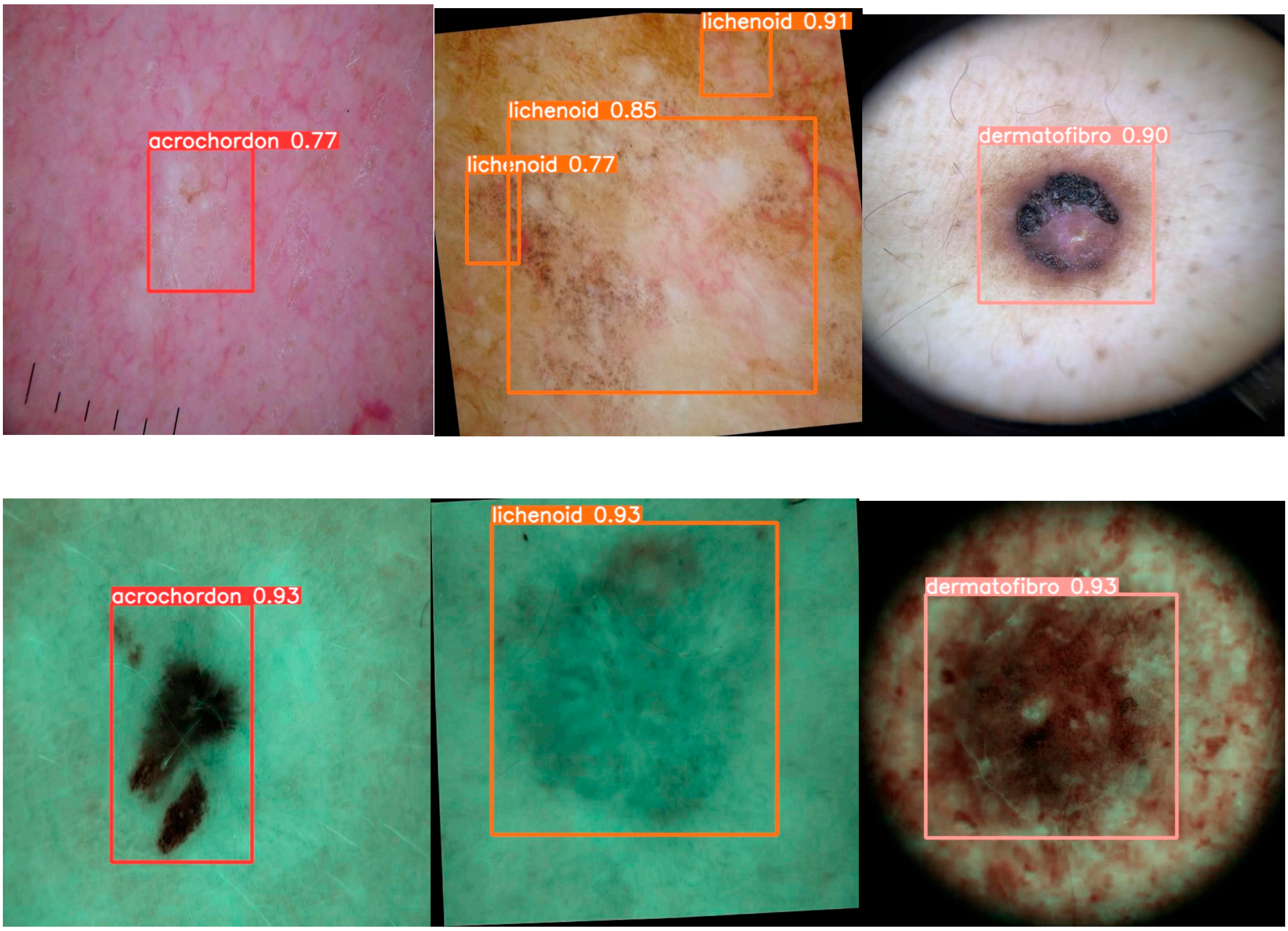

Figure S16. Comparison of WLI and SAVE results highlighting the contrast between the normal and the SAVE images.

S4. Evaluation of SAVE using VCE against WLI and NBI Olympus Endoscope

The valuation of the SAVE algorithm is comprehensively assessed through its performance metrics, specifically the Structural Similarity Index Metric (SSIM), pixel signal to noise ratio (PSNR) and entropy, which provide quantitative insights into the algorithm's effectiveness in image reproduction. At first SSIM between the simulated and the real WLI of the Olympus endoscope can be compared with the SAVE image from the HSI conversion algorithm developed in this study. SSIM can be defined as the amount of similarities between the simulated and the real image. The values range between 0% to 100% where 100% means the images are completely the same and 0% means the images are completely different. It is measured for the images that are reconstructed from a base image. Therefore, this parameter fits our criteria of comparison. Similarly, the WLI image of the VCE can also be compared with the SAVE images obtained from the HSI conversion algorithm. Figure S17 shows the SSIM for both the Olympus images and the VCE images. It can be seen that the Olympus images have a better SSIM rate with an average of 94.27% while the VCE has a comparatively lesser. Still, the average SSIM for VCE was found to be around 90%. This is because for NBI the CIEDE 2000 color calibration was possible for the traditional endoscope with the real NBI. After all, there was a reference real NBI image available. But for VCE no such reference is available. So, the same calibration that was

done for the NBI images of the Olympus was done for VCE. Even though without any reference the algorithm achieved a SSIM of 90%. It can also be seen that the top three highest achieved SSIM values was from the VCE of 96%. From this, we can infer that the results of the study are accurate. In this study 50 randomly chosen VCE images were used for calculating the SSIM. However, by increasing the number of images the SSIM can be significantly improved. Table 3 shows the SSIM comparison of twenty randomly chosen images in VCE and Olympus endoscope.

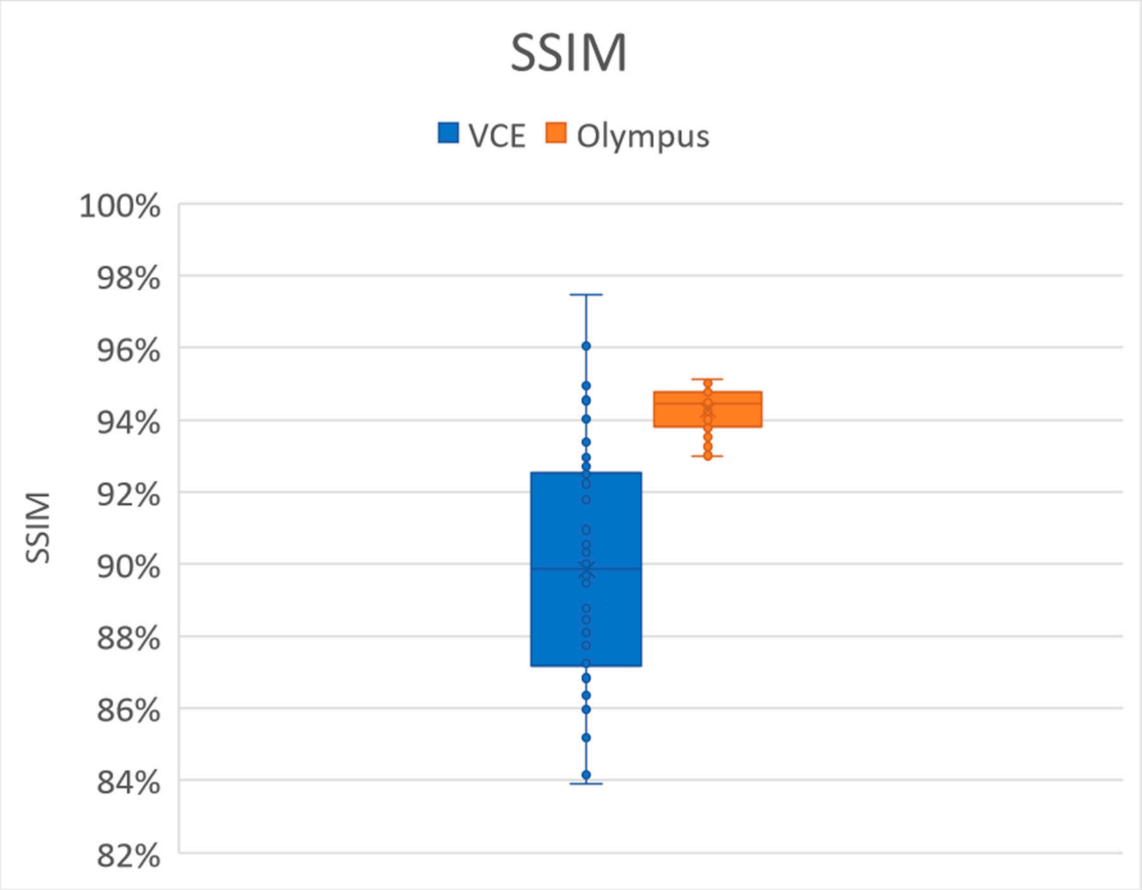

Figure S17. Comparison of SSIM between the simulated NBI images and the WLI images of VCE and Olympus

Table S1. SSIM of twenty randomly chosen images in VCE and Olympus endoscope.

Index	SSIM in VCE	SSIM in Olympus
1	0.925984794	0.94883467
2	0.944862636	0.948624384
3	0.928211385	0.94554478
4	0.966524653	0.937769205
5	0.92933774	0.941602499
6	0.958101429	0.945524249
7	0.939473779	0.935284698
8	0.936779292	0.933442268
9	0.872748906	0.938402164
10	0.873266671	0.936314054
11	0.891602213	0.934205211
12	0.944929655	0.949261138
13	0.805573811	0.941545835

14	0.90568673	0.946374234
15	0.83298981	0.945601764
16	0.912076221	0.932277471
17	0.936323837	0.930091206
18	0.792020729	0.939289678
19	0.936141641	0.932559509
20	0.903283862	0.935927389
Avg.	90.680%	93.992%

The second criterion that was used to evaluate the algorithm developed in this study was entropy. The entropy was also calculated similarly to the SSIM. The difference in entropy between the WLI images obtained from the Olympus endoscope is compared with the SAVE images simulated from the HSI conversion algorithm. In image processing, entropy might be used to classify textures, a certain texture might have a certain entropy as certain patterns repeat themselves in approximately certain ways. In the context of the paper low entropy means low disorder, low variance within the component. Therefore, lower the entropy better reproduction of the image is obtained. The difference in entropy between the WLI images obtained from VCE is compared with the SAVE images from the HSI-NBI conversion algorithm. Figure 18 shows the entropy difference in Olympus endoscope and VCE. As it can be seen from Table 4 the entropy difference in both the VCE and the Olympus endoscope have similar values. The average entropy difference in VCE was 1.17% while the average difference in the Olympus endoscope was 0.37%. However, in VCE the majority difference was found to be caused by only one image (image number 11). If we remove that image the entropy difference value is just 0.03% which is better than the Olympus endoscope. Table 4 shows the entropy comparison of the WLI and SAVE images in Olympus and VCE endoscope of twenty random images.

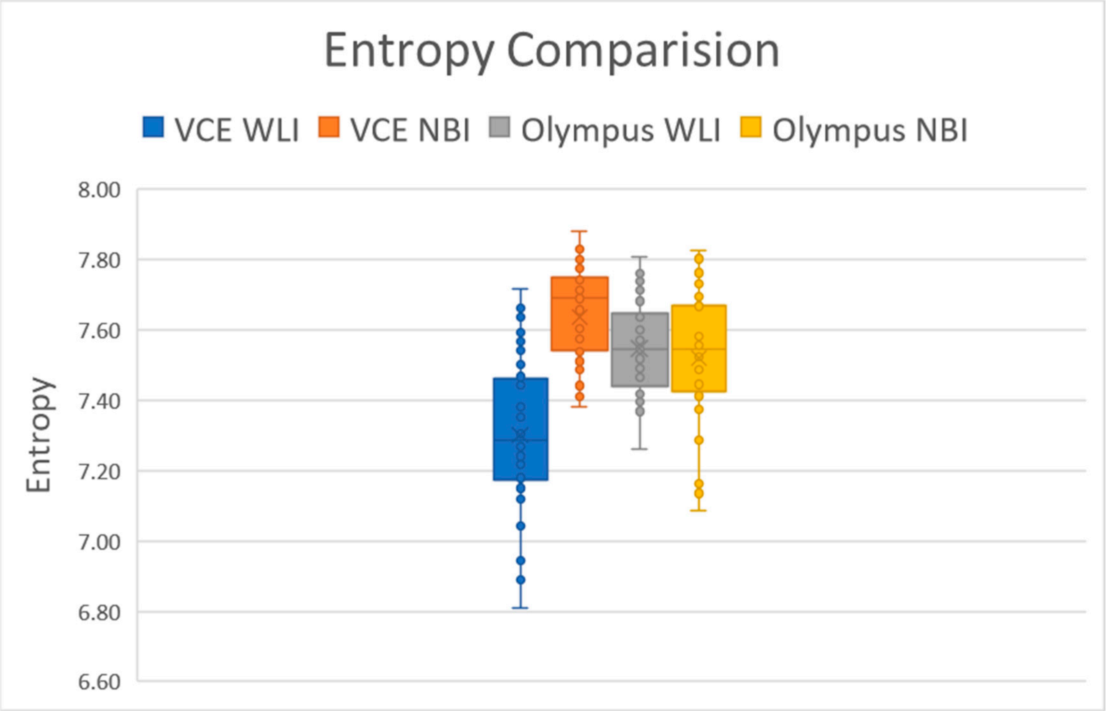

Figure S18. Comparison of entropy between the simulated NBI images and the WLI images. (a) Entropy for Olympus Endoscopy while (b) shows the entropy for the VCE camera.

Table S2. Entropy comparison of the WLI and NBI images in Olympus and VCE endoscope

Index	VCE		Endoscope		Difference in VCE	Difference in Olympus
	WLI	NBI	WLI	NBI		
1	7.04162	7.213345	7.60896	7.576404	0.024387	-0.004298
2	7.14502	7.220075	7.56380	7.494327	0.010504	-0.009271
3	7.37728	7.396152	7.65457	7.522734	0.002557	-0.017526
4	7.35948	7.278474	7.41264	7.283070	-0.011008	-0.017791
5	7.30720	7.363200	7.63545	7.680663	0.007663	0.005886
6	7.25352	7.350173	7.57114	7.546219	0.013325	-0.003167
7	6.88228	7.182308	7.53630	7.557263	0.043593	0.002773
8	7.25791	7.345877	7.71332	7.804702	0.012119	0.011708
9	6.95063	6.713150	7.56143	7.669351	-0.034167	0.014072
10	7.04094	7.134570	7.57816	7.676892	0.013297	0.012860
11	7.21094	6.575100	7.52565	7.372113	-0.088178	-0.020827
12	7.21771	7.219500	7.43596	7.422078	0.000247	-0.001871
13	7.58770	7.112978	7.57403	7.671311	-0.062566	0.012680
14	7.17281	7.184375	7.55415	7.667614	0.001612	0.014797
15	7.23312	7.210437	7.63470	7.693241	-0.003137	0.007608
16	7.14741	7.384973	7.71922	7.729203	0.033237	0.001291
17	7.28176	7.259479	7.75930	7.761126	-0.003061	0.000234
18	7.18062	6.801497	7.74727	7.801845	-0.052799	0.006994
19	7.26419	7.290983	7.74532	7.825175	0.003688	0.010205
20	7.59704	7.555495	7.736022	7.807877	-0.005469	0.009203
Avg	7.2254647	7.189607	7.61332	7.628160	-0.47%	0.19%

PSNR is typically utilized in the context of image compression algorithms as a parameter to evaluate the quality of the reproduced image. In a comparison of quality that is comparable to that of the SSIM, the PSNR values of twenty randomly selected WLI images and their SAVE equivalents are measured. The plot of the PSNR for each of the twenty images is displayed in Figure 19. The PSNR of the VCE images came in at an average of 28.0216 db, while the PSNR of the Olympus images was 27.8819 db. Table 5 shows the comparison of PSNR of the twenty randomly chosen images in Olympus and VCE.

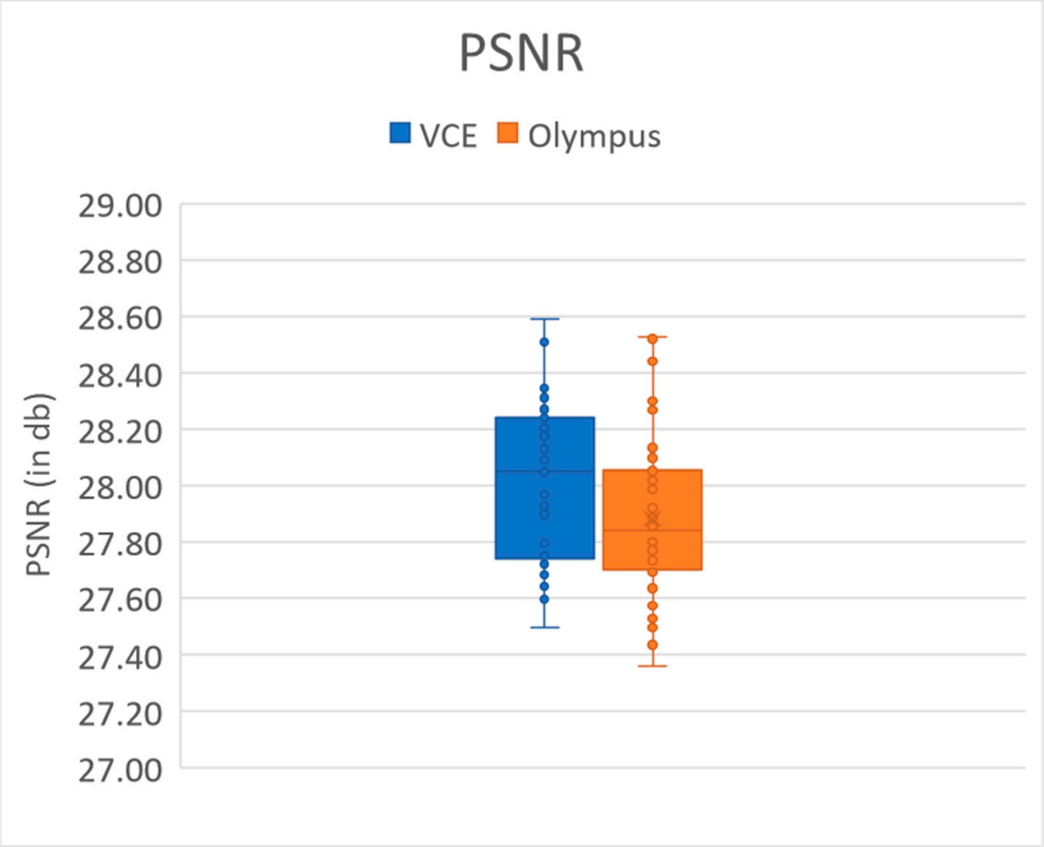

Figure S19. Comparison of PSNR of the twenty randomly chosen images in Olympus and VCE

Table S3. Comparison of PSNR of the twenty randomly chosen images in Olympus and VCE

Index	PSNR of Olympus images	PSNR of VCE images
1	27.5737493	28.51448883
2	27.4460567	27.72258326
3	27.46222796	27.66114567
4	27.36380762	27.49849716
5	27.82132106	27.67697859
6	27.49987858	28.1142441
7	27.78251246	27.90179575
8	27.71866018	27.6486571
9	27.78163772	27.65547302
10	27.63666623	28.0393251
11	27.58337722	28.20507339
12	27.70590967	27.99117518
13	27.53574671	28.26982928
14	27.68741185	27.9901207
15	27.74701762	28.26732897
16	27.81360865	27.7055983
17	27.87955844	27.90082759
18	27.71300104	28.50043856
19	27.73324857	27.68852332
20	28.02414942	27.68115621

Avg.	27.67547735	27.931663

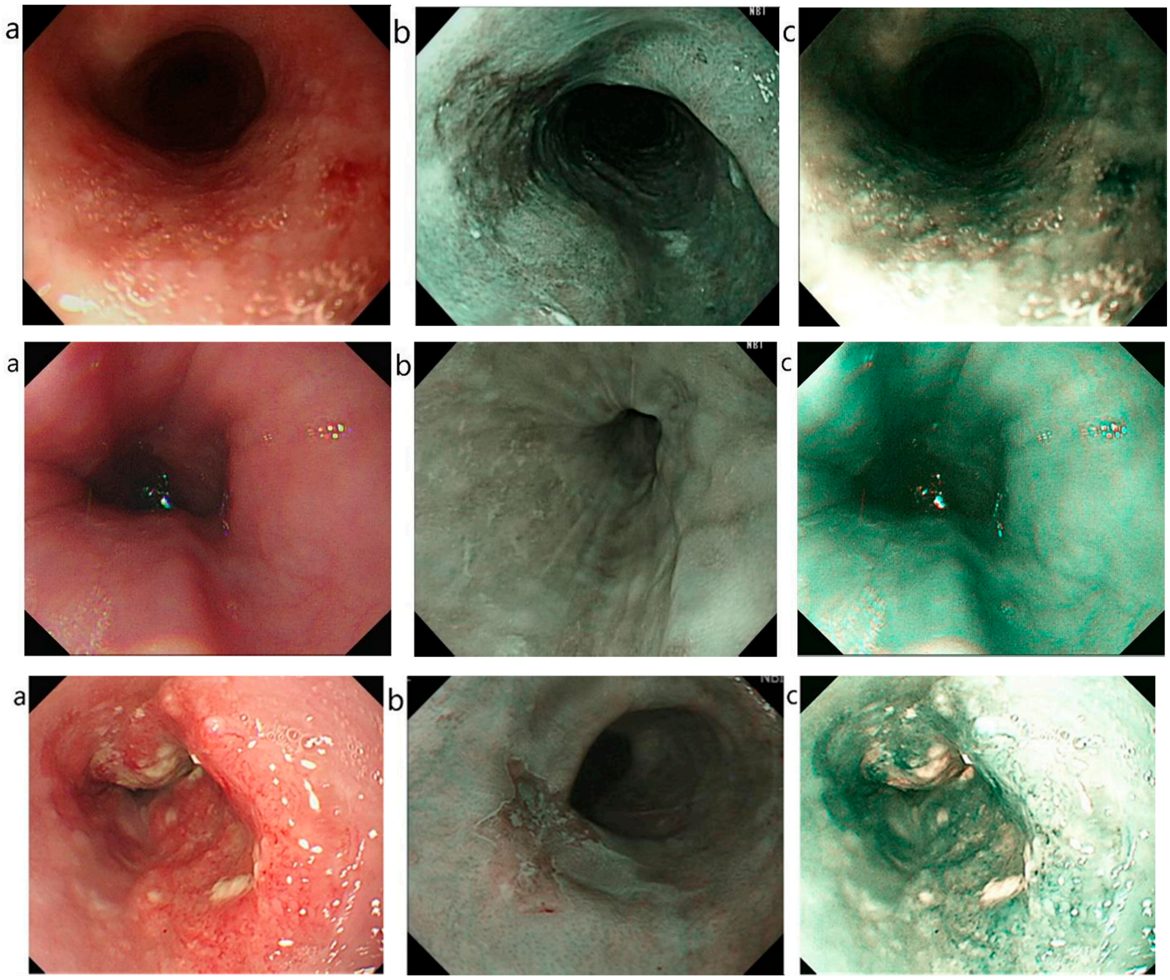

Figure S20. Endoscopic using three imaging techniques. (a) WLI, (b) NBI, (c) SAVE

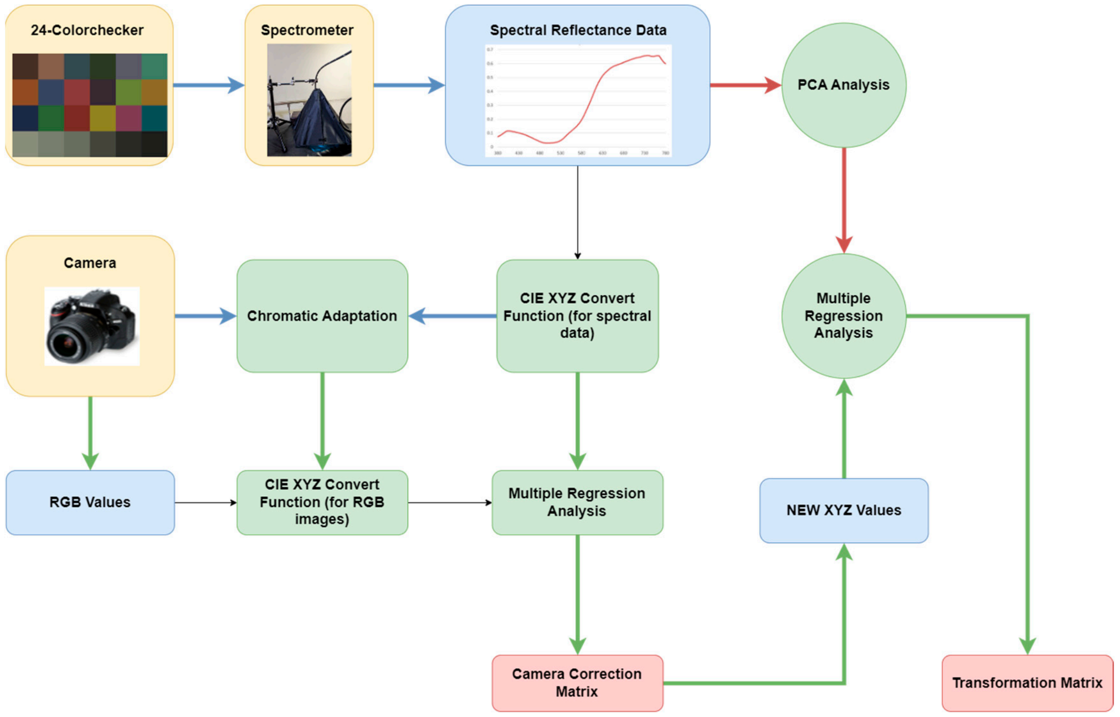

Figure S20. VIS-HSI imaging algorithm

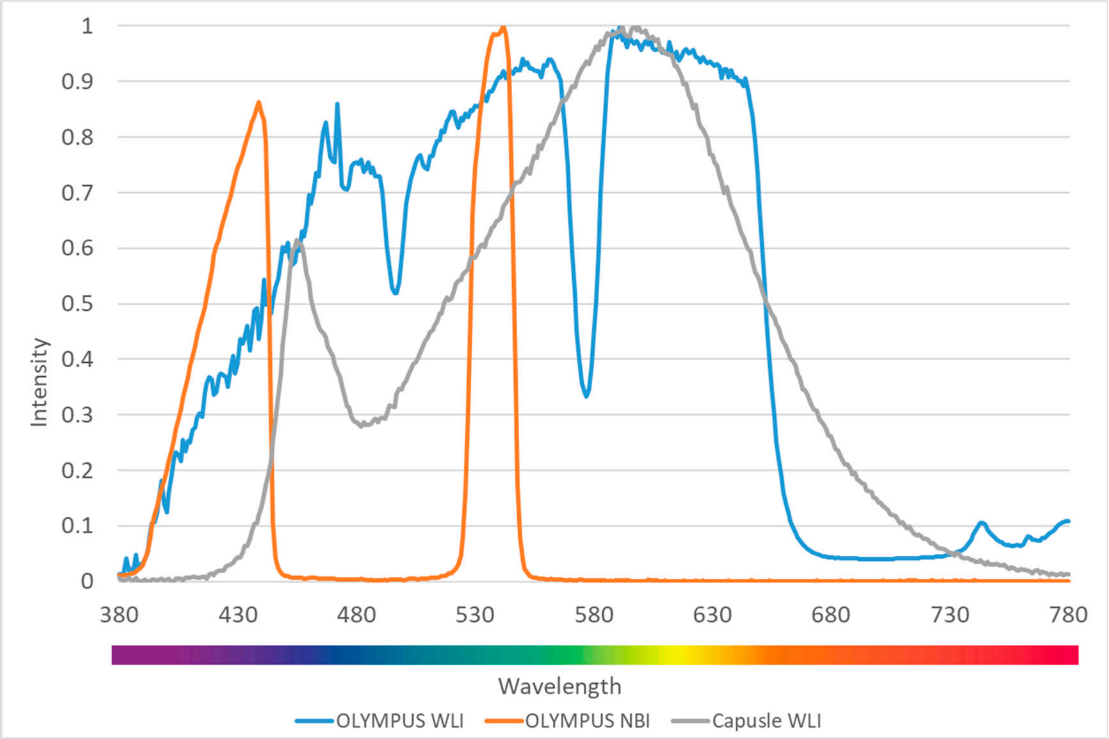

Figure S21. The lighting spectrum difference between the Olympus WLI, Olympus NBI, and the Capsule WLI.

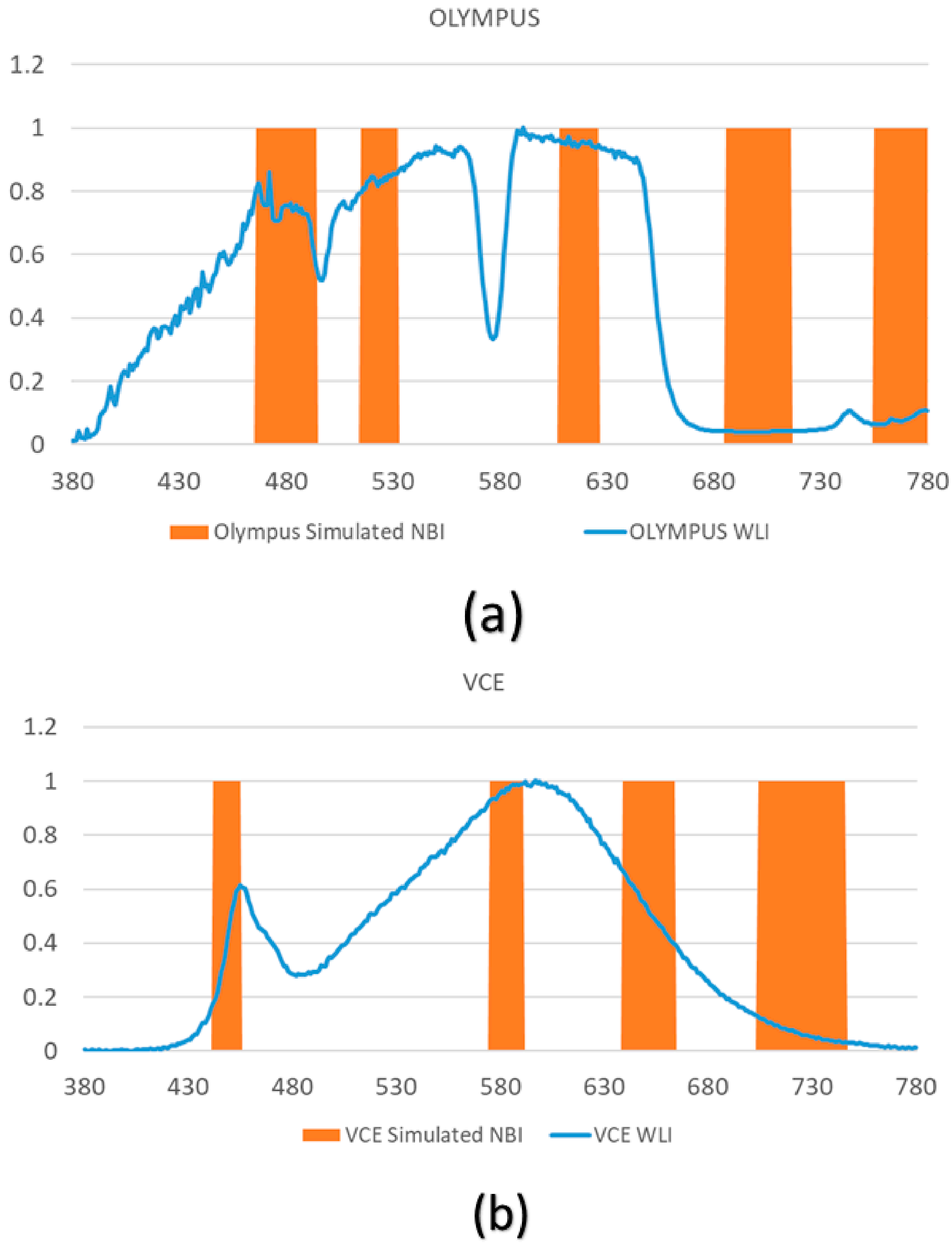

Figure S22. The Difference between the Olympus SAVE and the VCE simulated NBI lighting. (a) shows the difference between the Olympus SAVE and Olympus WLI. (b) shows the difference between VCE SAVE and VCE WLI images.

S5. Statistical Analysis

We conducted a paired statistical analysis of the F1-score differences between SAVE and WLI across our five YOLO variants to quantify the consistency and reliability of the observed performance gains. The mean improvement in F1 was 8.07%, with a 95% bootstrap confidence interval of [6.29%, 9.77%], demonstrating that the true average gain from SAVE is highly unlikely to fall below 6.3% or exceed 9.8%. To assess the median shift without assuming normality, we applied a Wilcoxon signed-rank test, which yielded a statistic of W = 0.00 and a p-value of 0.0625. Although this narrowly misses the $\alpha = 0.05$ significance threshold—reflecting the limited power of a five-sample comparison—every single paired difference was positive, providing strong evidence of a systematic

improvement. Moreover, the bootstrap interval does not include zero, reinforcing the practical significance of SAVE's effect. Taken together, these analyses confirm that our snapshot-based narrowband conversion yields a substantial, reproducible boost in YOLO's lesion-detection performance, while highlighting the value of larger-scale studies to further validate statistical significance.

Table S4. Comparison of F1-score of the different models.

Model	WLI F1 (%)	SAVE F1 (%)	Difference (%)
YOLOv11	57.67	65.73	8.06
YOLOv10	60.30	70.60	10.30
YOLOv9	66.50	71.50	5.00
YOLOv8	65.90	72.60	6.70
YOLOv5	57.30	67.40	10.10

Disclaimer/Publisher's Note: The statements, opinions and data contained in all publications are solely those of the individual author(s) and contributor(s) and not of MDPI and/or the editor(s). MDPI and/or the editor(s) disclaim responsibility for any injury to people or property resulting from any ideas, methods, instructions or products referred to in the content.
